# Gene Co-Expression Analysis Inferring the Crosstalk of Ethylene and Gibberellin in Modulating the Transcriptional Acclimation of Cassava Root Growth in Different Seasons

**DOI:** 10.1371/journal.pone.0137602

**Published:** 2015-09-14

**Authors:** Treenut Saithong, Samorn Saerue, Saowalak Kalapanulak, Punchapat Sojikul, Jarunya Narangajavana, Sakarindr Bhumiratana

**Affiliations:** 1 Bioinformatics and Systems Biology Program, School of Bioresources and Technology, King Mongkut’s University of Technology Thonburi, Thakham, Bangkhunthian, Bangkok, Thailand; 2 Systems Biology and Bioinformatics Research Group, Pilot Plant Development and Training Institute, King Mongkut’s University of Technology Thonburi, Thakham, Bangkhunthian, Bangkok, Thailand; 3 Center for Cassava Molecular Biotechnology, Faculty of Science, Mahidol University, Thungphayathai, Ratchathewi, Bangkok, Thailand; 4 Department of Biotechnology, Faculty of Science, Mahidol University, Thungphayathai, Ratchathewi, Bangkok, Thailand; 5 Department of Chemical Engineering, Faculty of Engineering, King Mongkut’s University of Technology Thonburi, Bangkok, Thungkhru, Bangmod, Bangkok, Thailand; Jaypee University of Information Technology, INDIA

## Abstract

Cassava is a crop of hope for the 21^st^ century. Great advantages of cassava over other crops are not only the capacity of carbohydrates, but it is also an easily grown crop with fast development. As a plant which is highly tolerant to a poor environment, cassava has been believed to own an effective acclimation process, an intelligent mechanism behind its survival and sustainability in a wide range of climates. Herein, we aimed to investigate the transcriptional regulation underlying the adaptive development of a cassava root to different seasonal cultivation climates. Gene co-expression analysis suggests that *AP2-EREBP transcription factor* (*ERF1*) orthologue (D142) played a pivotal role in regulating the cellular response to exposing to wet and dry seasons. The *ERF* shows crosstalk with gibberellin, via *ent*-Kaurene synthase (D106), in the transcriptional regulatory network that was proposed to modulate the downstream regulatory system through a distinct signaling mechanism. While sulfur assimilation is likely to be a signaling regulation for dry crop growth response, calmodulin-binding protein is responsible for regulation in the wet crop. With our initiative study, we hope that our findings will pave the way towards sustainability of cassava production under various kinds of stress considering the future global climate change.

## Introduction

Growing concern about food and energy shortage in this era has changed the view on plants. They are not only perceived as beautiful greenery but also as valuable carbohydrate reservoirs for all lives in the world. Cassava (*Manihot esculenta* Crantz*)* is a perennial crop plant whose underground roots contain up to 70–90 percent starch of total dry weight [1, 2]. As a starchy root crop, cassava is an important food source, similar to cereals (http://www.fao.org; http://www.fao.org/ag/magazine/0610sp1.htm) [3]. Its significance currently extends to be one of the main raw materials for green energy production [4, 5]. Another overt advantage of cassava is its high tolerance to harsh environment [1]. Cassava can grow in nutrient-poor soils where it is difficult to survive for the other staple crops. Nonetheless, the presently fluctuating climate tends to exceed the buffering limit of cassava, resulting in the decrease in cassava production, as experienced a few years ago [6, 7]. A rationale to prevent the severe loss of production might be to enhance the acclimation capacity of cassava plants to keep up with the future global climate change.

Cassava is believed to have an efficient acclimation mechanism, since it can grow in a wide range of climates and soil conditions across the tropical and sub-tropical regions. Despite being a perennial crop, cassava is grown twice a year during rains (wet crop) and post monsoon (dry crop) [8]. Cassava starches produced from the two seasonal crops are distinct in both yield and quality [8–11]. Season of planting is not the only factor determining the characteristics of cassava products; but climate conditions during the development and harvesting are also important effectors [10–12]. Since 1980s, in a series of studies, the response of cassava plants to the environment they had been exposed to has been investigated. Physiological processes that allow cassava to acclimatize to the surrounding climate, for example rhythm of stomatal closure and transpiration schedule, have been well described [13–17].

With the aid of advanced technology for intracellular measurement, observed physiological responses were shown to be closely regulated by the transcriptional regulation and plant hormone signaling [18–20]. The transcriptional responses of plants to varied climate and stresses were often involved in the plant hormone biosynthesis and metabolisms [18, 19, 21]. Plant hormones function as small signaling molecules that work with the transcription factors in gene transcriptional regulatory regime to modulate cellular responses. These small molecules mediate the activity of transcriptional factors, while the transcriptional regulation regulates the biosynthesis and metabolism of the molecules [22–24]. The examples of studies were the studies of drought stress response in *Arabidopsis thaliana*. In these studies, differentially expressed genes under the treatments were related to ABA and ethylene hormone biosynthesis and signaling pathways [19, 25]. In the same manner, investigation of salinity stress in rice showed the collaboration of the WRKY transcription factors and ABA hormone in the stress-response cascade [26, 27]. Extensive research has been conducted to reveal the role of each plant hormone in response to specific biotic and abiotic stresses as well as plant acclimation [21, 23, 28, 29]. However, plenty of evidence has indicated the association between hormones, called crosstalk, through the signal integrating transcription factor, such as KNOX [29], in response to a single stress circumstance [23, 29–31]. Ethylene, via ethylene responsive factor (ERF) and ABA hormones crosstalk, was reported in response to drought and submergence stresses in rice [32, 33]. The findings have demonstrated the scheme of the elaborated association matrix between hormone-hormone and hormone-TF (transcription regulator) in governing a cellular response to an exposure to any kinds of stress. Extensive studies of such regulation have been performed in *A*. *thaliana* and rice as model plants, as well as in other important crops such as tomato [34, 35] and wheat [36, 37]; however, there is no previous report on this aspect in cassava.

Attempts have been made to identify the stress-responsive genes in cassava [6, 38, 39], yet only in a few studies it has been reported how these gene candidates functioned cooperatively leading to plant’s resilience to the stresses they were exposed to. There are few reports on the transcriptional regulation relating to the adaptation of cassava to the season of planting. There is a closely related study by Sojikul *et al*. (2010) [40], where the genes relevant to cassava root development in two seasons of planting (wet and dry) were globally explored using cDNA-AFLP techniques. Here, in an extent to acquire the transcriptional regulation underlying the acclimation of cassava root to the plantation seasons, the highly expressed genes screened by cDNA-AFLP study [40] were subjectively selected for our gene co-expression analysis. Correlation statistics were calculated for the expression time-series of the genes taken from the study [40] followed by the network inferential method. The resulting gene co-expression network implied that plants employed different mechanisms in response to distinct kinds of stress and acclimating to the surrounding climates. Moreover, ethylene (via ERF (ethylene responsive factor)) and gibberellin (GA; via *ent*-Kaurene synthase, a key enzyme in GA biosynthesis) crosstalk is suggested to play a predominant role in cassava acclimation in distinct growing seasons.

## Materials and Methods

**Fig 1:**
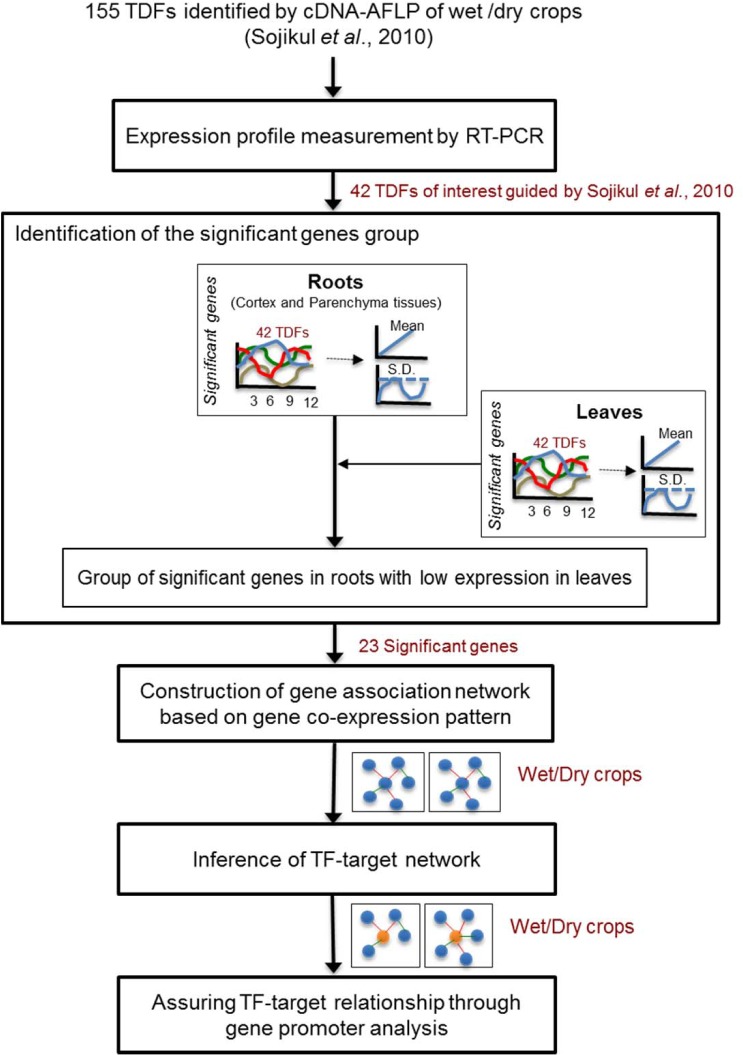
Overall methodology.

The seasonal acclimation of cassava roots was thoroughly studied through significantly expressed genes in roots of wet/dry crops. Transcriptional regulations in each crop were envisaged by the association networks of the significant gene sets. The methodology of the study was comprised of sequential steps, as illustrated in Fig 1.

### Plant materials and gene expression profile measurement

To study the transcriptional regulation related to distinct cassava root phenotypes of different seasonal cultivations, the expression of genes that were reported to be highly expressed in roots of Kasetsart 50 (KU50) variety, while showing low expression in their leaves [40], was a scope of our analysis. The time-series data measured by semi-quantitative RT-PCR of the genes of interest derived from transcript derived fragments (TDFs) were taken from cassava cDNA-AFLP analysis [40]. The employed TDFs and primer sequences that were developed from KU50 cassava variety were provided in [40] and also in GenBank database. In brief, the cDNA-AFLP analysis was employed to globally identify genes that were involved in cassava root growth in wet and dry season. The TDFs from two seasonal cultivations were measured in variety of tissues (*i*.*e*., roots (cortex and parenchyma) and leaves) throughout the growing process as summarized in S1 Fig. The two seasonal cultivations were grown at the end of rainy season (in September; dry season) and at the beginning of rainy season (in May; wet season); they were respectively denoted as ‘dry crop’ and ‘wet crop’ hereafter. The overly expressed genes under these conditions were further investigated the expression profiles using semi-quantitative RT-PCR. Pooled total RNA from the cDNA-AFLP samples (six plantlets) was used for expression analysis with TDF-specific primers [40]. Sixty ng of first-strand cDNA were used as templates in PCR. EF1α-specific primers were used in amplification to confirm uniformity of cDNA synthesis. The RT-PCR products were run on 1% agarose gel and visualized by ethidium bromide staining. Expression profiles were quantified using Quantity One image analysis software (GS800, BIORAD, Hercules, CA) [41]. After subtraction with lane-based background, intensity of bands of interest was quantified and normalized against the signal intensity of EF1α or 18S rDNA (see [40] for details of experimentation).

### Identification of the significant gene group

The expression profile was presumed to reflect the action of a gene as a mean of its role in the regulatory network. The significant gene set, which referred to the genes functioning under conditions of exposure, was identified based on the differential expression. In this work, the significant genes were determined from relatively different expressions, both in the level of abundance (Eq 1; significantly expressed at a certain stage of growth) and the pattern of expression (Eq 2; highly fluctuated throughout the growth period) across the time-series profiles.

$${\bar{X\;}}_{t}\left(G_{s}\right)\;\geq \;{\bar{X\;}}_{g}\left({\bar{X\;}}_{t}\left(G_{i}\right)\right)\;+\;{sd}_{g}\left({\bar{X\;}}_{t}\left(G_{i}\right)\right)$$ (1)

$${sd}_{t}\left(G_{s}\right)\;\geq \;{\bar{X\;}}_{g}\left({sd}_{t}\left(G_{i}\right)\right)\;+\;{sd}_{g}\left({sd}_{t}\left(G_{i}\right)\right)$$ (2)

As demonstrated in the equations, the significant genes (*G*_*s*_) were defined as a gene (*G*_*i*_) whose either average expression across the growth period (${\bar{X\;}}_{t}$) was relatively higher than the others, or whose expression pattern throughout the growth stages exhibited high variation (*sd*_*t*_). *g* and *t* denote the statistical analyses across gene-series and time-series, respectively. The calculation was conducted for gene expression measurements in all root and leaf tissues. Subsequently, the significant gene sets that were specific to root tissues were determined by excluding the significant gene set which also was present in leaf tissues. All of the statistical computation, herein, was carried out using built-in functions in Microsoft Excel.

### Construction of gene coexpression network

The relevance of genes functioning in the regulatory regime was inferred by their correlated expression patterns, according to the primary assumption of the co-expression analysis. The relationships of all gene pairs were assessed by Pearson correlation statistics, which basically quantified the similarity of the expression patterns in -1 to +1 scale coefficient, called *P* *earson* *C* *orrelation* *C* *oefficient* (*PCC*). The *PCCs* of each gene pair were calculated using Matlab R2015a for 64-bits Windows. The highly correlated expression patterns of gene pairs, with |*PCC*| ≥ 0.5, were subjectively included into the *gene co-expression network*. Cytoscape freeware (www.cytoscape.org/) [42] was employed for the network visualization.

The transcriptional regulatory network (*TF-target network*), which particularly describes the cooperation between the transcriptional regulatory factors (TFs) and their corresponding targeted genes (targets) in a regulatory control, was deduced from the previous gene association network. Among the correlated gene pairs, the TF-related gene associations were extracted to reconstruct the *TF-target network*. The constituent gene relationships in the *TF-target network* were passed to the upstream sequence analysis in the next step.

### Gene promoter analysis

Transcription factors always modulate the expression of target genes by acting on their promoter. *Cis*-acting regulatory elements in the upstream region of a gene, to which the specific transcriptional regulators bind (TFBS), are essential for achieving TF-driven expression. Accordingly, the relationships of TF-target gene pairs in the *TF-target network* were assured by conducting gene promoter analysis. Promoter regions of all genes were defined to cover up to 2000 bp upstream of the translation start site (TLS) based on cassava genome sequence version 4.1 in Phytozome database (www.phytozome.net/) [43]. The promoter sequences were then searched for TFBS and the corresponding TF using PlantPAN web-based tool (http://plantpan.mbc.nctu.edu.tw/) [44].

## Results and Discussion

Plant development, either at shoot apical meristem or at root part, is not only regulated by the genetically endogenous factors, but it is also equally influenced by the exogenous factors such as environmental climatic exposure [45–47]. Plants that are capable of sustaining their growth in a wide range of surrounding climates are believed to have an effective buffering system. As with cassava plant, how its roots acclimatize to the distinct water supply of the two seasons of planting is a challenging research question. Transcriptional regulation in response to cassava root acclimation, herein, was investigated in an extension of the previous findings by Sojikul *et al*. (2010). The original publication focused mainly on exploration of the genes involved in the storage root formation, with less analysis of root acclimation aspects. Through the cDNA-AFLP screening, they reported the group of genes that showed significant expression in cassava roots that underwent either wet or dry seasonal plantation [40]. The semi-quantitative expression time-series of these genes that were published along with the cDNA-AFLP study [40] were taken for our investigation.

### Ethylene and gibberellin may play significant roles in seasonal acclimation of cassava root development

**Fig 2:**
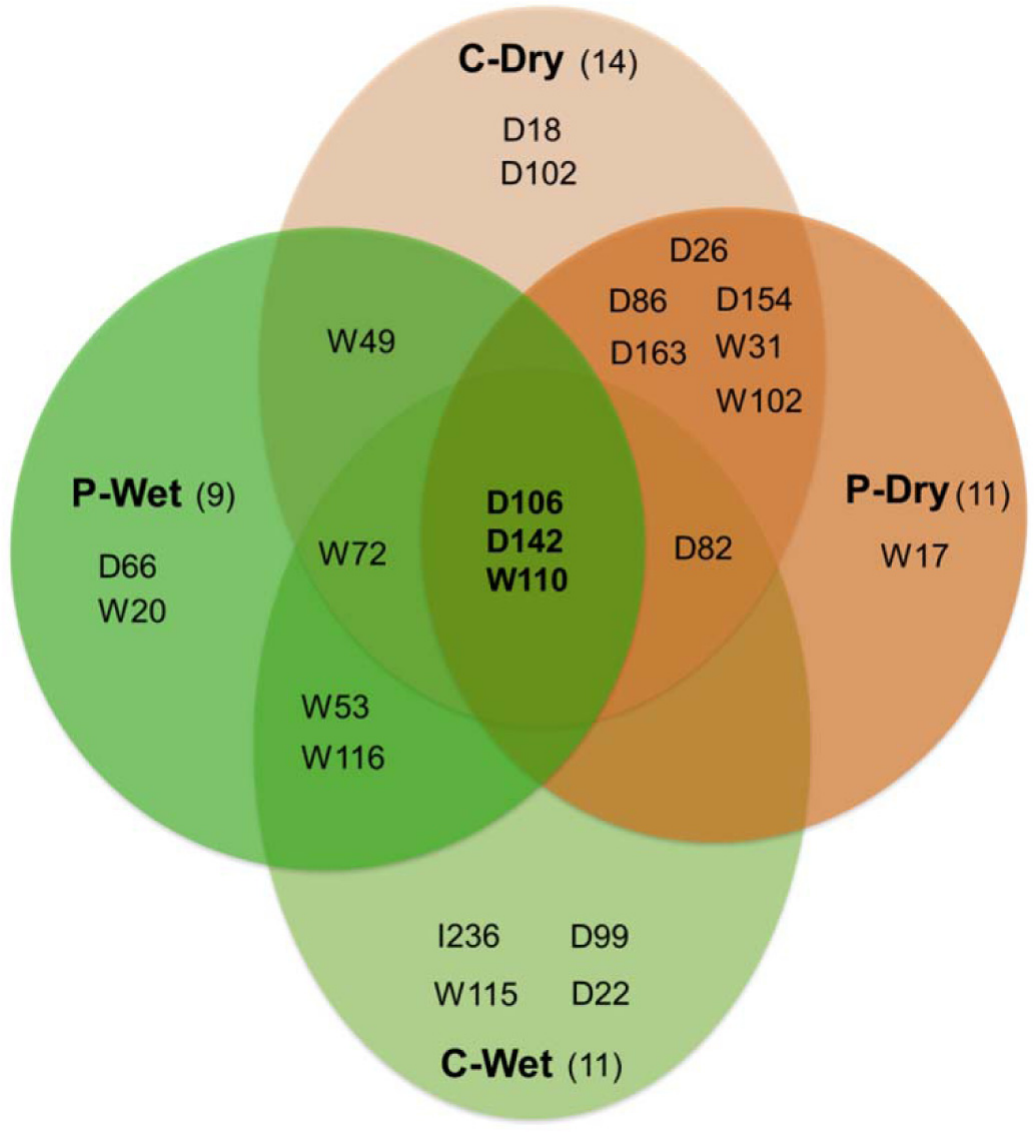
Significant genes that were differentially expressed during cassava root development in wet and dry seasons. C-Dry (light orange): cortex-dry, C-Wet (light green): cortex-wet, P-Dry (dark orange): parenchyma-dry, P-Wet (dark green): parenchyma-wet. The numbers in parentheses denote the total number of significant genes under each condition.

The 42 highly expressed genes suggested by the cDNA-AFLP experiment [40] were employed for the comparative analysis in order to acquire gene regulation relevant to root acclimation during development of cassava. The patterns of gene expression measured by the semi-quantitative method exhibited consistent profiles with those from the quantitative-based method (q-PCR), for example D83 (Calcium-dependent protein kinase), D154 (Hexose transporter), and D106 (*ent*-Kaurene synthase) (unpublished data). Stringent statistical analysis was applied to determine the significant expression of genes, showing either overt differential expression profiles or high level of expression, which were proposed to function under the studied conditions. The differentially expressed genes (DEGs) across plant developmental stages that were found to have a low profile in leaves were presumed to drive the regulation related to cassava root development; and they were also relevant to regulation underlying root acclimation when comparing them between the growing seasons. In total, 23 genes were identified as DEGs from the total of 42 genes. Fourteen (cortex tissue; C-Dry) and eleven (parenchyma tissue; P-Dry) genes were found to be differentially expressed in cassava roots of the dry crop, while eleven (cortex tissue; C-Wet) and nine (parenchyma tissue; P-Wet) genes were identified in roots of the wet crop (Fig 2). Majority of DEGs were transcription factors and regulatory genes involved in plant hormone biosynthesis and signaling. Among the DEGs, three genes, including D106 (*ent*-Kaurene synthase), D142 (AP2/EREBP transcription factor; ERF-1), and W110 (Senescence-associated protein DH), showed their significant expression in all root tissues from both crops. It implies the significant function of plant hormones, especially ethylene (D142) and gibberellin (D106), in modulating acclimation of cassava root during development under the growing conditions, while the senescence-associated protein (W110) may reflect the progress of aging process in developed roots. Predominance of ethylene and gibberellin hormones and signaling corresponds to extensive studies of the molecular responses of a plant to water stresses, including drought and flooding (via hypoxia circumstance). The influence of ethylene hormone in flooding stress has been shown to trigger gibberellin (GA) and abscisic acid (ABA) for plant development and response to stress, respectively [48]. Moreover, ethylene biosynthesis and signalling have also been shown to be provoked by drought [49].

In addition, our results showed that on the basis of significant gene sets, groups of genes were specifically expressed either in the root tissue or under a certain growth condition. Fig 2 demonstrates the genes significantly expressed in particular crops, either wet or dry, which function in a specific root tissue (cortex or parenchyma). Nine genes, consisting of D18 (nucleotide pyrophosphatase-like protein), D102 (mitotic checkpoint family protein), D26 (NADH dehydrogenase subunit 2, NADH dehydrogenase subunit 1), D86 (ubiquitin), D163 (hypothetical protein), D154 (hexose transporter), W31 (mitrocondrial citrate synthase precursor), W102 (S-adenosyl-L-methionine synthetase 1), and W17 (AtVOZ1 transcription factor) showed explicit expression in roots of the dry crop, whereas eight genes, including D66 (Myb domain protein 33 transcription factor), W20 (zinc finger transcription factor), W53 (catalase CAT1), W116 (calmodulin binding protein), I236 (WRKY DNA-binding protein 33), D99 (cinnamoyl CoA reductase), W115 (3-ketoacyl-CoA thiolase; acetyl-CoA acyltransferase), and D22 (UDP-glucuronosyltransferase) were expressed differentially in roots of the wet crop. Diverse significant gene sets in roots of the two crops indicated distinct transcriptional regulation underlying acclimation of cassava roots to different climates of the two growth conditions during the developmental process. With few common significant genes in the two crops, it is interesting to investigate how these significant genes orchestrated to modulate the acclimation of cassava root developed in the wet and dry cultivating seasons.

### Co-expression genetic networks demonstrated the distinct transcriptional regulation underlying cassava root development grown in different seasons

**Fig 3:**
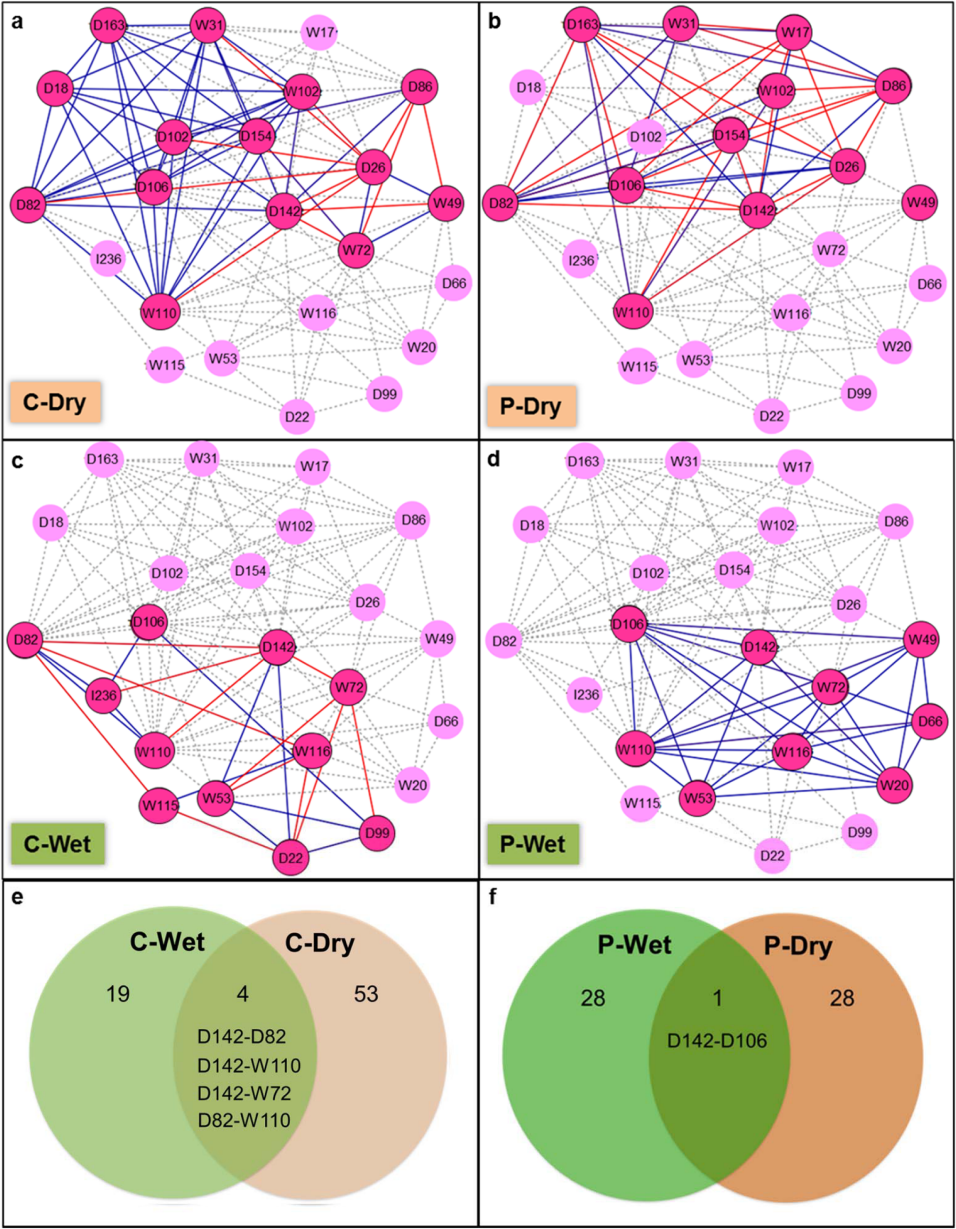
Gene co-expression networks of cassava root development in wet and dry growing seasons. (**a**) C-Dry: cortex-dry, (**b**) P-Dry: parenchyma-dry, (**c**) C-Wet: cortex-wet, (**d**) P-Wet: parenchyma-wet. The networks were comprised of 23 genes that showed significant expression under each growth condition. The highlighted nodes and edges (blue: positive relationship and red: negative relationship) represent the active gene and gene association, presumably functioning under the certain condition. (**e**) and (**f**) demonstrates the comparison of associated gene pairs between the two seasonal crops in root cortex and parenchyma tissues, respectively. The abbreviation code of a gene in the co-expression network could be found in S2 File.

While the phenotypic response of plants is always a result of sophisticated cooperation of genes, analysis in the previous section provided only the set of gene candidates playing a role under the observed conditions. Therefore, gene collaborative networks were derived from the co-expression profiles of the significant genes (so called *gene co-expression network*) to estimate the intracellular regulatory network in roots of wet and dry crops (see also Materials and Methods). The overall co-expression network of 23 significant genes from both crops was presented in Fig 3, where the highlighted darker colors represent the significant genes (circles) and gene-gene relationships (lines) presumably functioning under a particular condition (Fig 3A–3D). The resulting four co-expression networks (*i*.*e*. C-Dry (Fig 3A), P-Dry (Fig 3B), C-Wet (Fig 3C), and P-Wet (Fig 3D)) clearly exhibit the different transcriptional regulation occurring in root tissues (cortex (C) and parenchyma (P)) of wet and dry crops. Compared gene-gene relationships comprised the networks of both crops; more than 83 percent of relationships (C-Wet (19/23), C-Dry (53/57), P-Wet (28/29), and P-Dry (28/29)) were found to be unique for a growth condition, whereas only four and one gene associations were a common in the networks of root cortex (Fig 3E) and parenchyma (Fig 3F), respectively. It implies that the developing roots of cassava employed different gene regulatory systems to sustain their growth under different cultivation conditions. Moreover, the difference between co-expression networks of root cortex and parenchyma from one crop cultivation suggests the spatial-specific gene regulatory system that may provide a particular contribution in a regulatory regime.

The common associated gene-pairs may refer to a key player participating in the acclimation process of the developing roots. Interestingly, three of five common gene-gene relationships changed correlative function according to the cultivation conditions. D142 expression positively correlated with D82 and W110 in C-Dry; however, the transcription of these genes shows opposite relationships in C-Wet (Fig 3A and 3C). Also, the relationship of D142 and D106 in parenchyma tissue changed from positive in P-Wet to a negative manner in P-Dry (Fig 3B and 3D). The findings support the predominant role of ethylene signaling (via ethylene responsive factor, D142) as a modulator for cassava root acclimation to water availability at the early settlement of a plant, experienced in different growing seasons. Besides ethylene hormone, gibberellin hormone (GA) and sulfur metabolism are likely to be involved in this regulatory regime. The co-expression network demonstrates the ethylene and gibberellin signaling crosstalk in root parenchyma tissue (D142-D106; Fig 3B, 3D and 3F) and association of ethylene hormone and sulfur metabolism in root cortex tissue (D142-D82; Fig 3A, 3C and 3E). Evidence supporting the relevance of these regulatory factors in water stress and plant development has been reported in *A*. *thaliana* [50], as well as in other model plants such as tobacco [51]. Sulfur metabolism might interplay with ethylene in transducing environmental signal to trigger the regulatory network, resulting in the phenotypic response of a plant. Our co-expression network shows that the role of sulfur and ethylene is more dominant in the dry season crop (Fig 3A and 3B). Correspondingly, a recent study demonstrated the involvement of ethylene and sulfur in regulatory response of Medicago roots and nodules to drought. It showed that water deficiency provoked the downregulation of ethylene and methionine (a part of sulfur assimilation) biosynthesis pathways [49]. Incorporating the previous knowledge [49, 52, 53] with our results, the observed relationship of ethylene and sulfur metabolism could be explained as follows: sulfite reductase (D82) facilitates the assimilation of sulfur to produce cysteine which can be converted to methionine, a substrate for ethylene biosynthesis through S-adenosyl-L-methionine synthetase 1 (W112). The produced ethylene, in turn, has a regulatory interaction with glutathione (GSH), another product of cysteine, whose level evidently shows interrelationship with the water availability. To gain more insight into the regulatory regime behind cassava root acclimation, associated gene pairs of a transcription factor (TF) were closely analyzed.

### Transcriptional regulatory network inferred bidirectional action of ethylene responsive transcription factor and GA-related genes in modulating acclimation of cassava root development in wet and dry growing seasons

**Fig 4:**
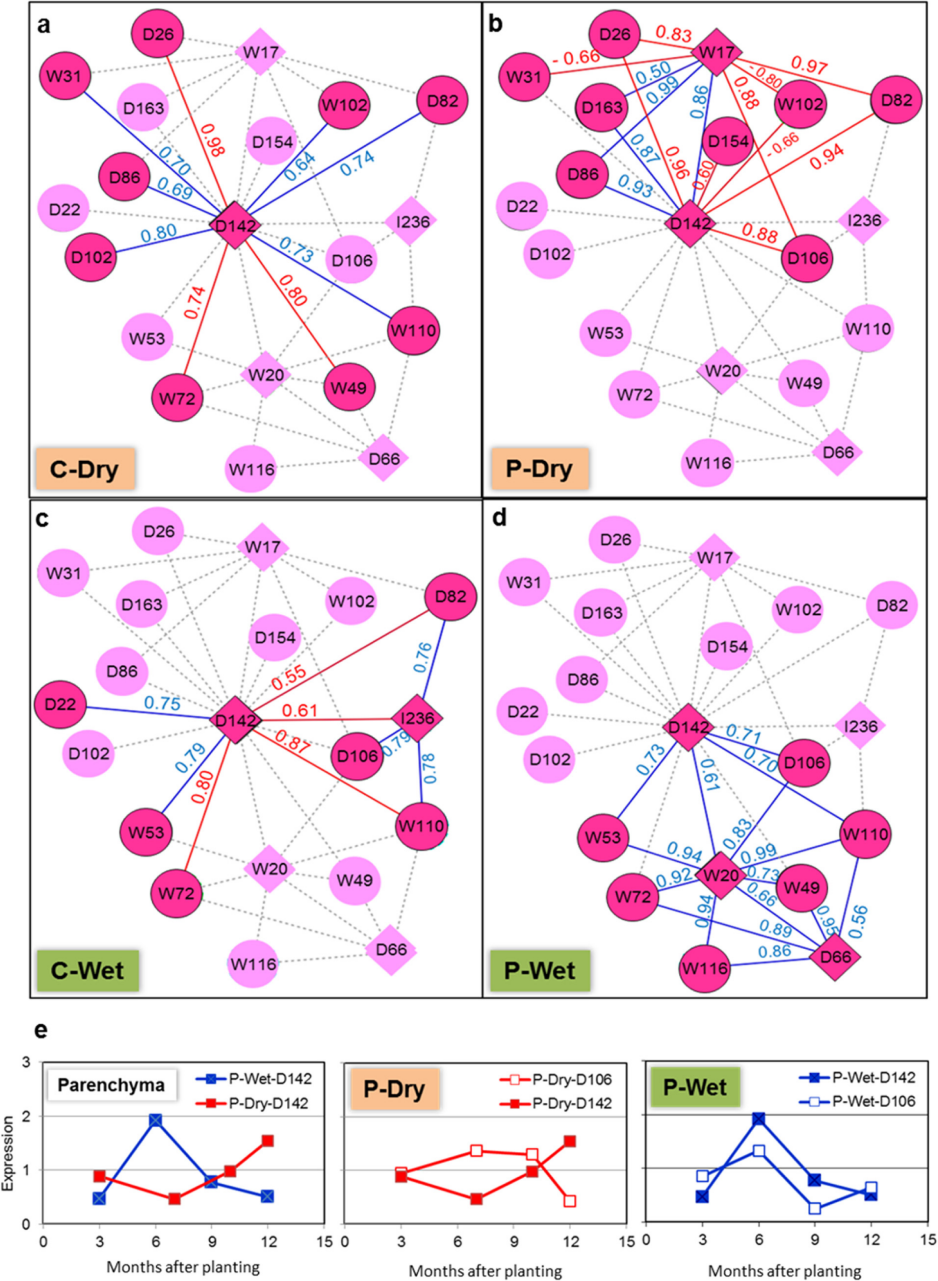
Transcription regulatory networks (TF-target network) of cassava root development in wet and dry growing seasons. (**a**) C-Dry: cortex-dry, (**b**) P-Dry: parenchyma-dry, (**c**) C-Wet: cortex-wet, (**d**) P-Wet: parenchyma-wet. The highlighted nodes (diamond: transcription factor genes and circle: target genes) and edges (blue: positive relationship and red: negative relationship) represent the active gene and gene association, presumably functioning under the condition. The numbers aligned with the highlighted edges denote the PCC of the expression profile for the associated gene pair. (**e**) demonstrates the expression profiles of D142 and D106 which are the key regulating factors in the transcription regulatory networks

Transcriptional regulatory network (TRN), which describes the association of transcription factors (TF) and their regulated target genes, was deduced from the previous gene co-expression networks. Overall, the TRNs of cassava roots herein consisted of the relationships of five TF genes that show significant expression under at least one condition in the study: D142 (AP2/EREBP transcription factor; ERF-1), D66 (Myb domain protein 33 transcription factor), W17 (AtVOZ1 transcription factor), W20 (zinc finger transcription factor), and I236 (WRKY DNA-binding protein 33). Fig 4 demonstrates the TRNs of cassava roots grown in dry (Fig 4A and 4B; dark color highlighted) and wet (Fig 4C and 4D; dark color highlighted) seasons, where the TFs were denoted as a diamond. As observed, the TRNs of dry-crop roots explicitly differ from those of wet-crop roots, especially in parenchyma root tissue. It indicates that the regulatory difference in acclimation processes of the two crops is not only present at gene-gene association, but also occurs in the TF-target system. In the TRNs regime, the hypothesized role of D142 in modulating root acclimation is more pronounced. It is the only TF that was significantly expressed in root tissues under all conditions, and it was positioned at the center of the network regulating a large number of target genes. Our work might be another study supporting the primary function of ethylene hormone in triggering and modulating the downstream signaling hormones/pathway in response to water stress as proposed by many collective evidence in *A*. *thaliana* and other model plants [48, 54]. Furthermore, the results suggested that the similar regulation is conserved in cassava root.

Focusing on the TRNs of root parenchyma where starch is fully accommodated, ethylene and gibberellin signaling crosstalk was hypothesized to play a great role in shaping the succeeding regulatory systems to respond to the climatic exposure throughout the root development period. Bidirectional relationship between D142 and D106 in P-Dry and P-Wet infers the adaptive regulatory gene association that allows cassava roots to be capable of developing under distinct climates (Fig 4B and 4D). The downstream regulation, which was influenced by the changed harmony of ethylene signaling and gibberellin metabolism, may reflect the buffering regulatory systems employed in the roots of each crop. Though the downstream genes are functionally diverse, the developed TRNs provide a seed idea of the underlying biological processes involved in cassava root acclimation under water-restricted growth. In the dry season crop, D142 and D106 were antagonistically expressed in root parenchyma (P-Dry; Fig 4B), suggesting the inhibitory effect of ethylene signal on the gibberellin biosynthesis. This hormone crosstalk presumably delivers the signal for acclimatory responses by working with a phloem-specific transcription factor (W17; AtVOZ1 transcription factor) and a key enzyme relating to sulfur sensing pathway (D82; Sulfite reductase). The relevance of sulfur signaling to water deprivation has been described for various plants, for example maize and *A*. *thaliana* [52, 53, 55]. For wet seasonal crop, D142 and D106 genes have a co-expression pattern which indicates the synergistic role of these hormones under such conditions. The TRN in Fig 4D demonstrates completely different regulation downstream of ERF-GA hormone crosstalk from that present in dry crop roots. The hormonal signal was not carried over by sulfur sensing pathway, but it was related to the two transcription factors, W20 (zinc finger transcription factor) and D66 (Myb domain protein 33 transcription factor), in cooperation with calmodulin-binding protein (W116) and senescence-related genes (W110). Calmodulin-binding protein is a group of proteins functioning in transduction of Ca^2+^ signal, a second messenger, to modulate the physiology of plants in response to both biotic and abiotic stresses [56, 57]. It has been found to act with ethylene in response to hypoxia under flooding conditions in *A*. *thaliana* [58, 59] by working together with many transcription factors, including MYB family (*e*.*g*. MYB2 [60]) and zinc-finger family TFs [56, 61]. For example, Ca^2+^/calmodulin-mediated ethylene signaling was reported to modulate MYB2 TF in response to low-oxygen conditions (hypoxia or anoxia, a circumstance referring to flooding stress) [54, 60]. In a similar manner, calmodulin-binding protein (W116) might be a part of acclimation regulatory regime inside cassava roots planted in wet season when rainfall is stably fed at an early period of planting.

The adaptive response of plant’s growth to water stress often shows the participation of ABA hormone in the signaling regulatory cascade in addition to ethylene and gibberellin. Surprisingly, our TRN demonstrated the remarkable role of ethylene and gibberellin crosstalk in modulating cassava root development under water stresses; however, the action of ABA could not be observed. A potential explanation of this finding might be as discussed by Verelst *et al*. (2010) [50]. Though the three phytohormones are involved in the same cascade in order to co-modulate the growth under water stresses, they in fact progress their function separately in different organs. The study on the adaptive growth under drought stress in *A*. *thaliana* reported that ABA targets only at the mature cells (mature leaves), while ethylene and gibberellin are responsible for the expansion and division of younger cells (young leaves and shoot) [50, 62]. Lack of ABA action in our TRNs that were developed from the significantly expressed genes in cassava roots may thus be caused by the fact that the sampled root cells were not a cell of action for ABA.

**Fig 5:**
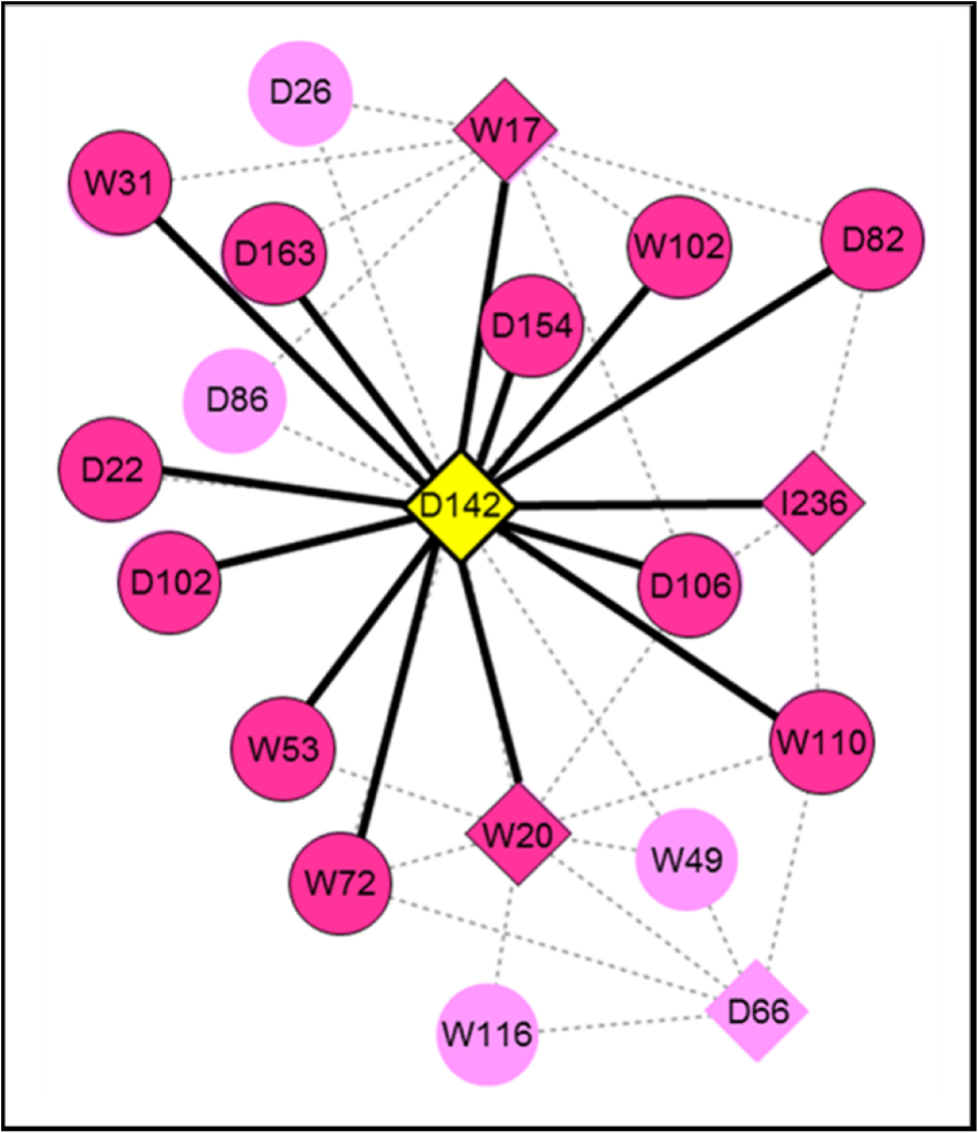
*Cis*-regulatory element analysis in the upstream region of the D142-target genes. The highlighted nodes and edges marked the associated TF-target pair whose transcriptional regulatory relationship could be supported by the finding of TF-binding site (TFBS).

To assure the association of TF and target genes on which the proposed regulatory mechanism relies, gene promoter analysis was carried out to explore *cis*-acting regulatory elements essential for their transcriptional control. Two-kilobase upstream sequences of 23 genes in the TRNs were subjectively analyzed by PlantPAN (http://plantpan.mbc.nctu.edu.tw/) [44]. The results were discussed as emphasized on D142 (see also S1 File) because of the appearing prominent role in the TRNs and its relatively clearer gene annotation than the other transcriptional factors. Fig 5 demonstrates the *cis*-elements-confirmed relationships of D142 and its target genes marked with highlighted color. While almost all predicted gene associations of D142 supported the direct regulation of D142 on the target gene transcription, D26 and D86 genes, whose expression patterns were highly correlated with those of D142 (PCC_D142-D26_ = -0.96 and PCC_D142-D86_ = 0.93), lacked in ERFBP-domain for the transcription factor on their promoters. One explanation of the un-confirmed relationships, herein, could be the indirect transcriptional control between D142 and these two target genes that may at least require another mediator for conveying the regulatory signal. In regard to the co-expression of D26 and D86 with D142, it was only found in roots of the dry crop where the phloem-specific transcription factor (W17) was significantly expressed. Therefore, the results may imply that D142 modulates transcription of D26 and D86 genes partly through the phloem-specific transcription factor.

**Fig 6:**
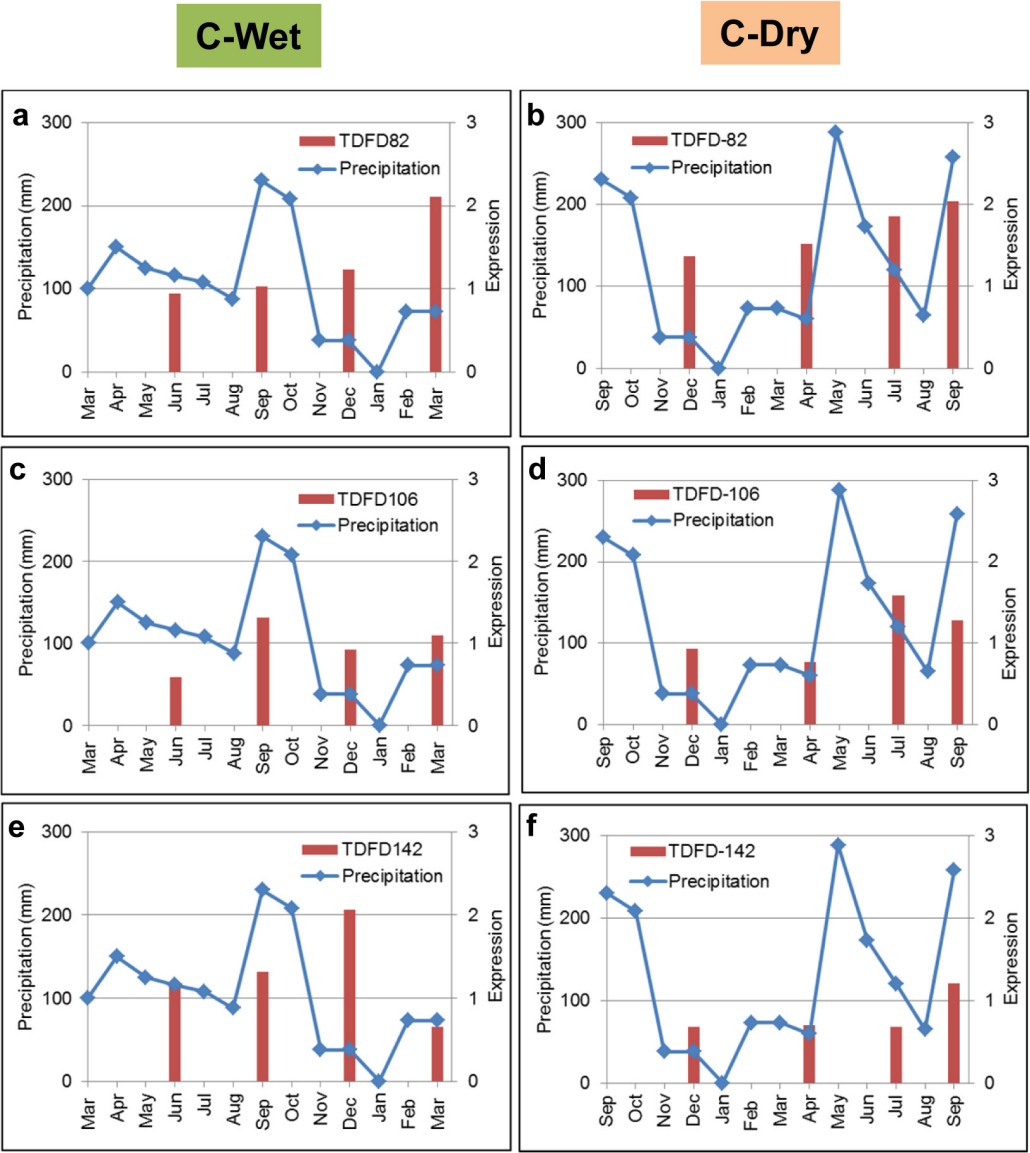
Expression profiles of key genes in the cortex transcriptional regulatory network. The gene expression (red bar) was superimposed onto the precipitation curve (blue line) for comparison: (**a-b**) D82, (**c-d**) D106 and (**e-f**) D142.

**Fig 7:**
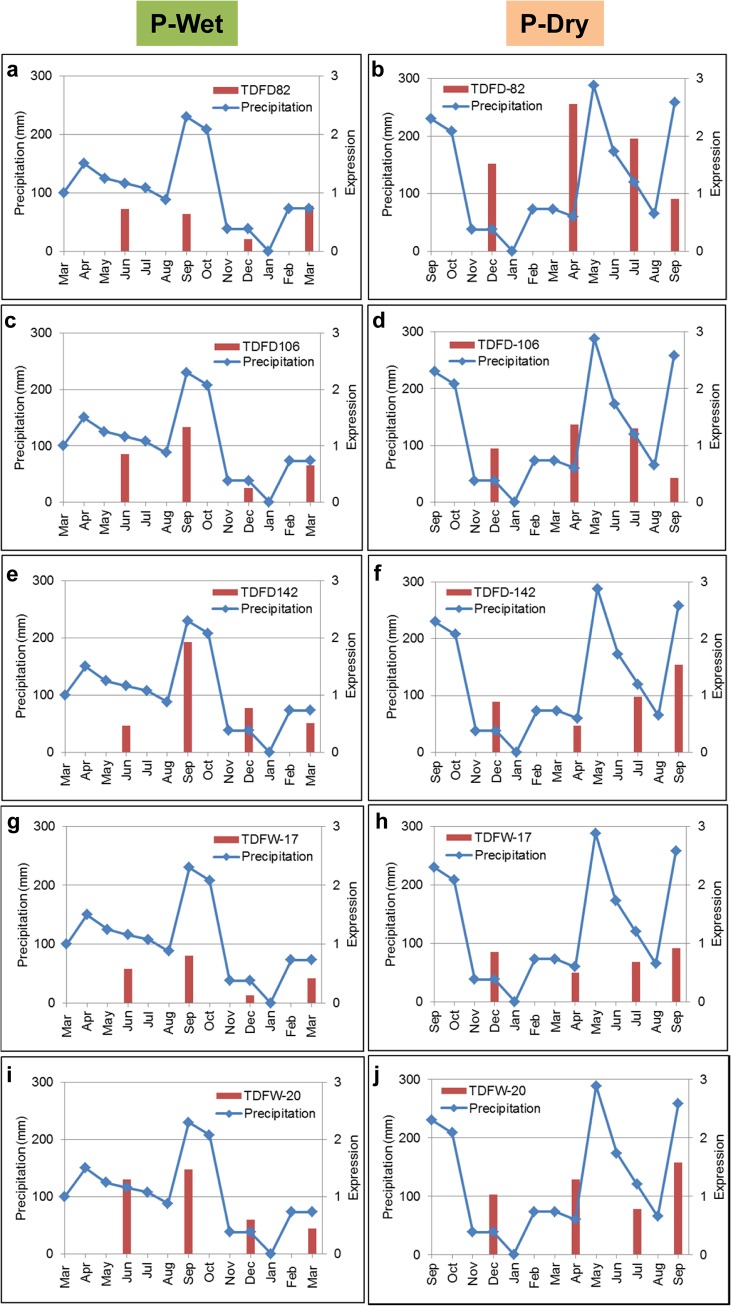
Expression profiles of key genes in the parenchyma transcriptional regulatory network. The gene expression (red bar) was superimposed onto the precipitation curve (blue line) for comparison: (**a-b**) D82, (**c-d**) D106, (**e-f**) D142, (**g-h**) W17 and (**i-j**) W20.

Transcriptional regulatory networks highlighted the crosstalk between ethylene and GA-related genes. They also demonstrated its regulatory function in triggering the downstream regulation for root acclimation to seasons of planting. The remaining mystery regarding the proposed function is how this regulatory mechanism allows plants to sense the varying climatic exposure. For the two plantation seasons, early rainy season (wet) and the end of rainfalls (dry), the major climatic factor is the quantity of water available for growth at the beginning of plant settlement. The expression profiles of the key genes in TRNs were thus investigated against the rain precipitation throughout the growing periods (Figs 6 and 7). The results showed that D142 was highly expressed in both seasonal crops whereby the patterns of expression were tightly influenced by the amount of precipitation. Conversely, D106 and other transcription factor genes exhibited subtle expression levels and their profiles were highly correlated to D142 expression rather than directly affected by the surrounding climate. It suggests that cassava root perceived the environmental signal via ethylene responsive factor (D142) which later crosstalked with gibberellin metabolism (via D106) to induce the downstream genes responsible for the acclimation process.

## Conclusions

**Fig 8:**
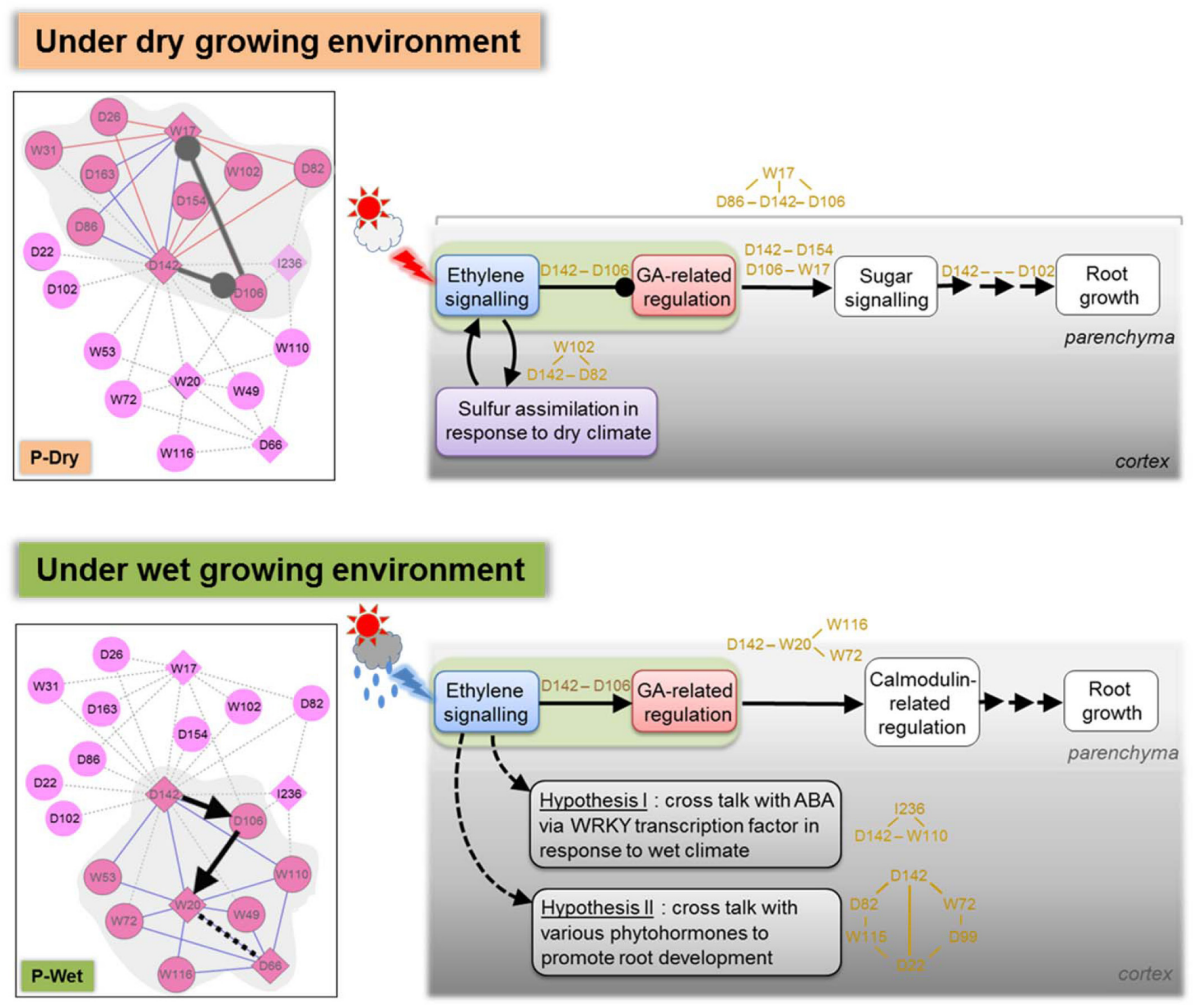
Scheme of the proposed model.

To elucidate the transcriptional regulation governing the cassava root acclimation in the two plantation seasons, the transcriptional regulatory networks of cassava roots were conducted to represent the intracellular gene regulation throughout plant cultivation. The results indicate distinct manners of regulation responsible for root acclimation to the climate. Different manners of regulations could be observed in both root cortex and parenchyma tissues. Although the TRNs of root cortex help support the different manners of regulation found in root parenchyma of the two crops and show a spatial-dependent regulatory system, the information is not sufficient to draw a final conclusion. The model of cassava root acclimation to wet and dry season was thus proposed mainly according to the results of analyses in root parenchyma, as shown in Fig 8. The model describes a hypothetical scenario of transcriptional control underlying the cassava root acclimation in different seasons of planting. Ethylene responsive transcription factor (ERF; D142) plays a role in environmental signal perception. As observed, its expression was strongly influenced by the exposed surrounding climate (Fig 7). The expressed ERF, as a result, collaborates with the GA-related genes, functioning as the core modulator of the downstream regulation. The distinct correlation manners of ERF and GA-related genes, negatively correlated in P-Dry and inversed in P-Wet, lead to a shift in the group of genes responsible for the transcriptional control regulation. In the dry crop, the ethylene and gibberellin crosstalk is likely to acclimatize root development under limited water rainfall via sulfur assimilation and sucrose signaling-related regulation. In the wet crop, the hormone crosstalk triggers the calmodulin-related regulation in response to the abundance of water in rainy season.

The highly elaborated transcriptional regulation of cassava root acclimation that is involved in various regulatory systems, including hormones and other small signaling molecules as well as TF-driven gene expression, may be a trade-off for the great adaptability of cassava roots to the varying and broad range of environmental climates. The combination of signaling molecules and hormones could generate a great number of signaling codes to precisely deliver the exposed condition to a cell where the signals are conveyed and employed to modulate distinct regulatory gene groups depending on the conditions. This strategic mechanism could provide a huge variety of cassava responses to a particular exposed climate, as reported for other plants [21, 23]. The good compensation of complexity and flexibility of cassava transcriptional regulatory network presented in this work would be one of many jigsaws to decipher the secret of the effective acclimation regulatory system in cassava, resulting in highly tolerant plants.

## Abbreviations

### Codes of annotated genes in the co-expression network (also provided in S1 File)

**Table:** 

D22	UDP-glucuronosyltransferase
D26	NADH dehydrogenase subunit 2, NADH dehydrogenase subunit 1
D29	GAG-POL precursor/Retrotransposon protein
D30	Protein kinase
D63	Non-green plastid inner envelope membrane protein
D67	RAB GTPase activator
D77	Cytosolic phosphoglucomutase
D78	H(+)-transporting ATPase
D80	Hypothetical protein containing TPR domain
D82	Sulfite reductase
D83	Calcium-dependent protein kinase
D86	Ubiquitin
D90	Catalytic/methionine gamma-lyase
D94	Putative succinyl-CoA synthetase
D95	Zinc-finger DNA binding protein
D99	Cinnamoyl CoA reductase
D100	Ferritin-1, chloroplast precursor
D102	S-adenosyl-L-methionine; synthetase 1
D106	ent-Kaurene synthase
W72	Glycoside hydrolase family 28 protein/polygalacturonase (pectinase) family protein
D154	Hexose transporter
D163	Hypothetical protein
W17	Transcription factor AtVOZ1
W20	Zinc finger protein (*Camellia sinensis*)
W27	Receptor-like protein kinase-like protein (*Oryza sativa*)
W28	Sinapyl alcohol dehydrogenase (*Populus tremuloides*)
W31	Mitrocondrial citrate synthase precursor (*Citrus junos*)
W38	Non-intrinsic ABC protein (*Nicotiana benthamiana*)
W49	Hypothetical protein ZeamMp158 (*Zea mays*)
W51	Protein translation factor SUI1 homolog (*Salix bakko*)
W53	Catalase CAT1 (*Manihot esculenta*)
D142	AP2/EREBP;Transcription factor; ERF-1
W102	S-adenosyl-L-methionine synthetase 1 (*Daucus carota*)
W107	NADK1 (NAD kinase 1); NAD+ kinase/ NADH kinase/ calmodulin binding
W110	Senescence-associated protein DH (*Z*. *mays*)
W115	3-ketoacyl-CoA thiolase; acetyl-CoA acyltransferase (*Cucumis sativus*)
W116	EDA39 (embryo sac development arrest 39); calmodulin binding
W120	Inositol polyphosphate 5-phosphatase, putative
W124	UBX domain-containing protein
I236	WRKY DNA-binding protein 33
D22	UDP-glucuronosyltransferase
D26	NADH dehydrogenase subunit 2, NADH dehydrogenase subunit 1
D29	GAG-POL precursor/Retrotransposon protein
D30	Protein kinase
D63	Non-green plastid inner envelope membrane protein
D67	RAB GTPase activator
D77	Cytosolic phosphoglucomutase
D78	H(+)-transporting ATPase
D80	Hypothetical protein containing TPR domain
D82	Sulfite reductase
D83	Calcium-dependent protein kinase
D86	Ubiquitin
D90	Catalytic/methionine gamma-lyase
D94	Putative succinyl-CoA synthetase
D95	Zinc-finger DNA binding protein
D99	Cinnamoyl CoA reductase
D100	Ferritin-1, chloroplast precursor
D102	S-adenosyl-L-methionine; synthetase 1
D106	ent-Kaurene synthase
W72	Glycoside hydrolase family 28 protein/polygalacturonase (pectinase) family protein
D154	Hexose transporter
D163	Hypothetical protein
W17	Transcription factor AtVOZ1
W20	Zinc finger protein (*Camellia sinensis*)
W27	Receptor-like protein kinase-like protein (*Oryza sativa*)
W28	Sinapyl alcohol dehydrogenase (*Populus tremuloides*)
W31	Mitrocondrial citrate synthase precursor (*Citrus junos*)
W38	Non-intrinsic ABC protein (*Nicotiana benthamiana*)
W49	Hypothetical protein ZeamMp158 (*Zea mays*)
W51	Protein translation factor SUI1 homolog (*Salix bakko*)
W53	Catalase CAT1 (*Manihot esculenta*)
D142	AP2/EREBP;Transcription factor; ERF-1
W102	S-adenosyl-L-methionine synthetase 1 (*Daucus carota*)
W107	NADK1 (NAD kinase 1); NAD+ kinase/ NADH kinase/ calmodulin binding
W110	Senescence-associated protein DH (*Z*. *mays*)
W115	3-ketoacyl-CoA thiolase; acetyl-CoA acyltransferase (*Cucumis sativus*)
W116	EDA39 (embryo sac development arrest 39); calmodulin binding
W120	Inositol polyphosphate 5-phosphatase, putative
W124	UBX domain-containing protein
I236	WRKY DNA-binding protein 33

### Codes of studied conditions

**Table:** 

C-Dry	cortex tissue of a cassava root grown in dry season
P-Dry	parenchyma tissue of a cassava root grown in dry season
C-Wet	cortex tissue of a cassava root grown in wet season
P-Wet	parenchyma tissue of a cassava root grown in wet season

## Supporting Information

S1 Fig Scheme of plant sample collection (TIF)

S1 File Abbreviation codes of genes in the co-expression network. (PDF)

S2 File *Cis*-regulatory element in the upstream region of the D142-target genes identified by PlantPan. (PDF)
