# Supplementary figures and images for: Gene Co-Expression Analysis Inferring the Crosstalk of Ethylene and Gibberellin in Modulating the Transcriptional Acclimation of Cassava Root Growth in Different Seasons
Source: PLoS One. 2015 Sep 14;10(9):e0137602. doi: 10.1371/journal.pone.0137602 (PMC4569563; doi:10.1371/journal.pone.0137602)

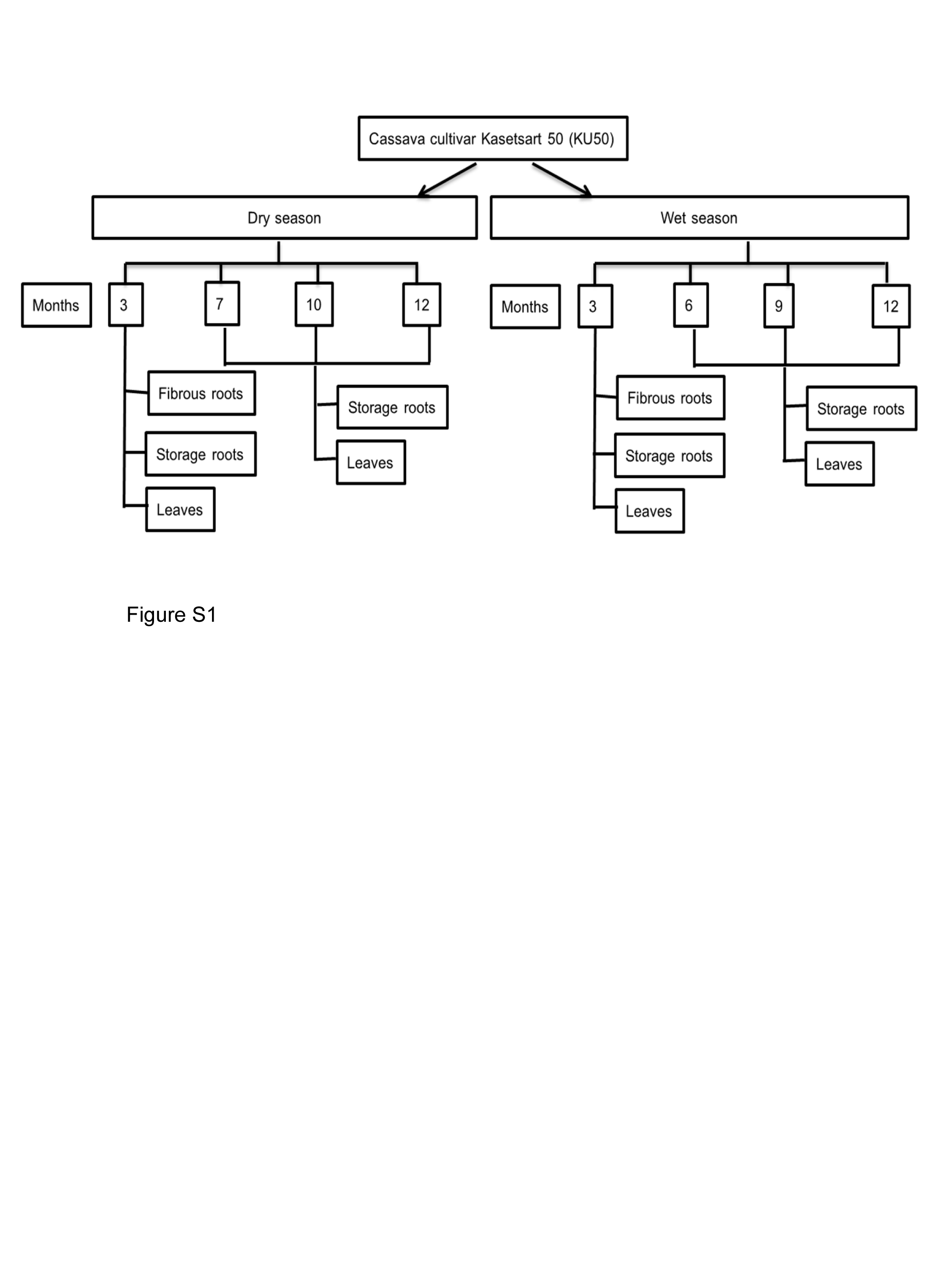

Supplement: S1 Fig — (TIF) [file pone.0137602.s001.tif]
