# Supplementary material for: Gene Co-Expression Analysis Inferring the Crosstalk of Ethylene and Gibberellin in Modulating the Transcriptional Acclimation of Cassava Root Growth in Different Seasons
Source: PLoS One. 2015 Sep 14;10(9):e0137602. doi: 10.1371/journal.pone.0137602 (PMC4569563; doi:10.1371/journal.pone.0137602)
Supplement: S1 File — (PDF) [file pone.0137602.s002.pdf]

Supplementary data

D22	UDP-glucuronosyltransferase
D26	NADH dehydrogenase subunit 2, NADH dehydrogenase subunit 1
D29	GAG-POL precursor/Retrotransposon protein
D30	Protein kinase
D63	Non-green plastid inner envelope membrane protein
D67	RAB GTPase activator
D77	Cytosolic phosphoglucomutase
D78	H(+)-transporting ATPase
D80	Hypothetical protein containing TPR domain
D82	Sulfite reductase
D83	Calcium-dependent protein kinase
D86	Ubiquitin
D90	Catalytic/methionine gamma-lyase
D94	Putative succinyl-CoA synthetase
D95	Zinc-finger DNA binding protein
D99	Cinnamoyl CoA reductase
D100	Ferritin-1, chloroplast precursor
D102	S-adenosyl-L-methionine ; synthetase 1
D106	ent-Kaurene synthase
W72	Glycoside hydrolase family 28 protein/polygalacturonase (pectinase) family protein
D154	Hexose transporter
D163	Hypothetical protein
W17	Transcription factor AtVOZ1
W20	Zinc finger protein (Camellia sinensis)
W27	Receptor-like protein kinase-like protein (Oryza sativa)
W28	Sinapyl alcohol dehydrogenase (Populus tremuloides)
W31	Mitrocondrial citrate synthase precursor (Citrus junos)
W38	Non-intrinsic ABC protein (Nicotiana benthamiana)
W49	Hypothetical protein ZeamMp158 (Zea mays)
W51	Protein translation factor SUI1 homolog (Salix bakko)
W53	Catalase CAT1 (Manihot esculenta)
D142	AP2/EREBP;Transcription factor ; ERF-1
W102	S-adenosyl-L-methionine synthetase 1 (Daucus carota)
W107	NADK1 (NAD kinase 1); NAD+ kinase/ NADH kinase/ calmodulin binding
W110	Senescence-associated protein DH (Zea mays)
W115	3-ketoacyl-CoA thiolase; acetyl-CoA acyltransferase (Cucumis sativus)
W116	EDA39 (embryo sac development arrest 39); calmodulin binding
W120	Inositol polyphosphate 5-phosphatase, putative
W124	UBX domain-containing protein
I236	WRKY DNA-binding protein 33
