# Supplementary material for: Gene Co-Expression Analysis Inferring the Crosstalk of Ethylene and Gibberellin in Modulating the Transcriptional Acclimation of Cassava Root Growth in Different Seasons
Source: PLoS One. 2015 Sep 14;10(9):e0137602. doi: 10.1371/journal.pone.0137602 (PMC4569563; doi:10.1371/journal.pone.0137602)

Supplemental data: Analysis of TF families on each target of 5 TFs

1. List of targets of TDFD66 (Myb domain protein 33)

Order	TF	Target
1	D66	W49
2	D66	W72
3	D66	W110
4	D66	W116
5	D66	W20

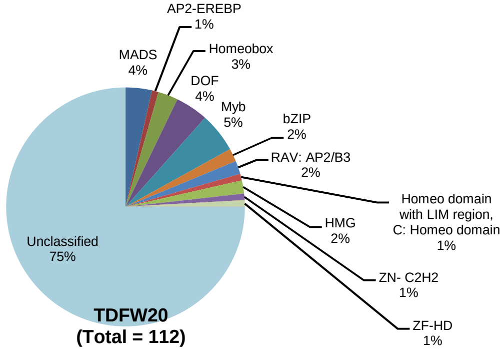

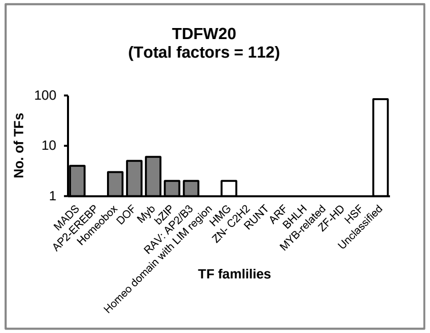

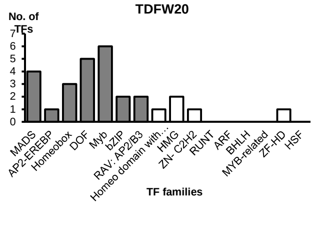

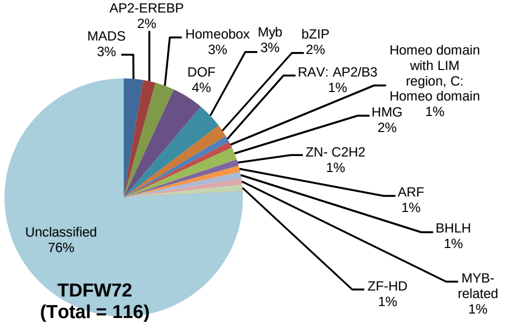

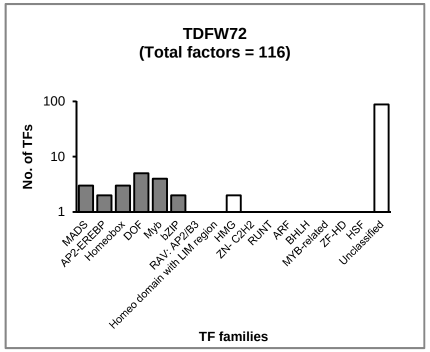

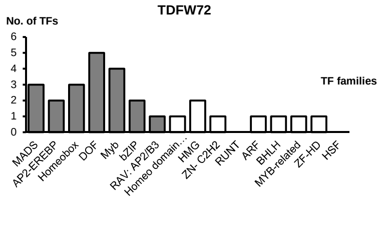

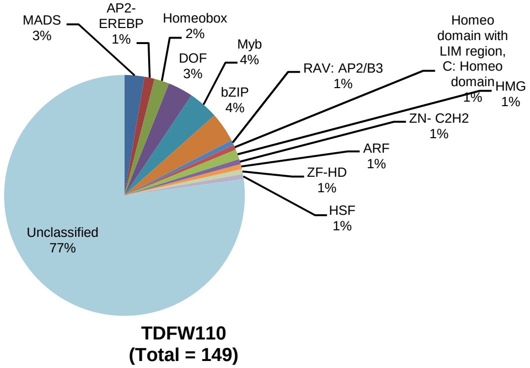

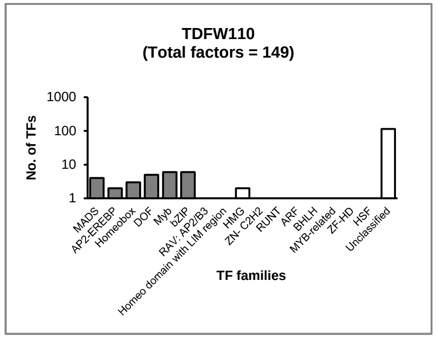

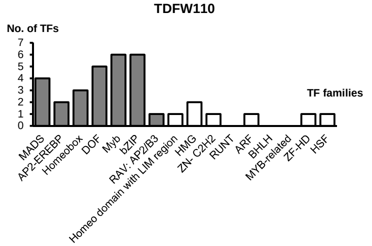

1. List of targets of TDFD66 (Myb domain protein 33)

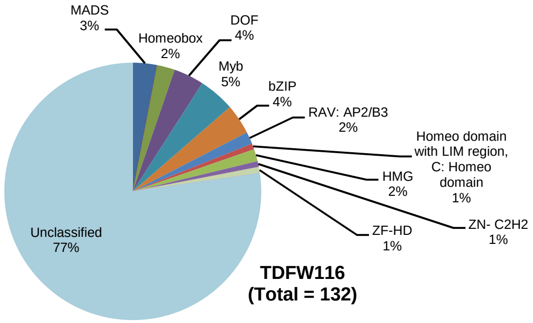

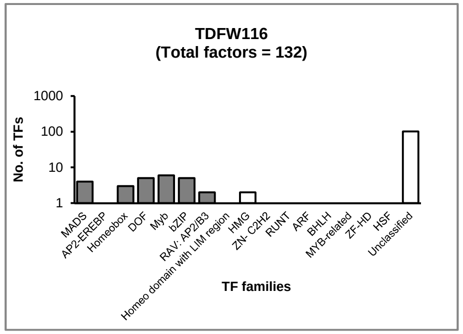

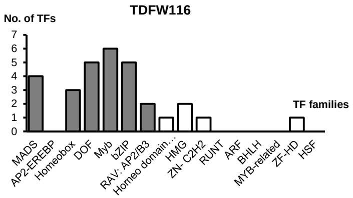

2. List of targets of TDFD142 (Ethylene responsive element binding protein)

Order	TF	Target
1	D142	D22
2	D142	D26
3	D142	D82
4	D142	D86
5	D142	D102
6	D142	D106
7	D142	D154
8	D142	D163
9	D142	W31
10	D142	W49
11	D142	W53
12	D142	W72
13	D142	W102
14	D142	W110
15	D142	I236
16	D142	W17
17	D142	W20

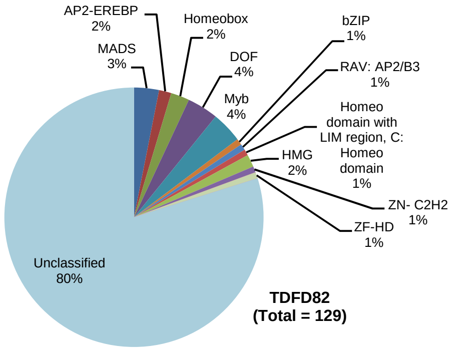

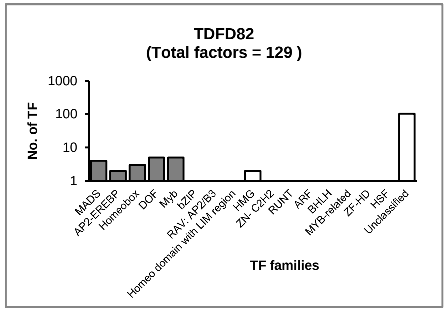

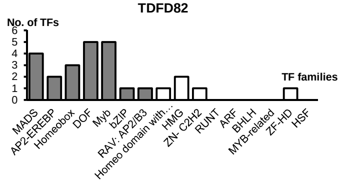

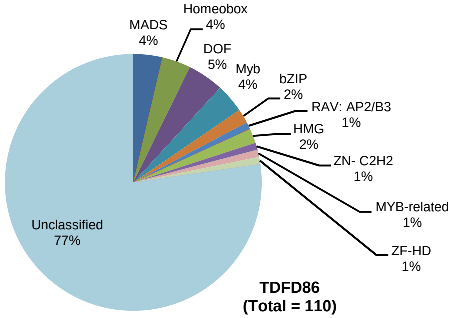

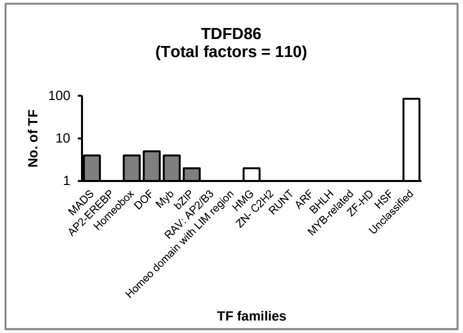

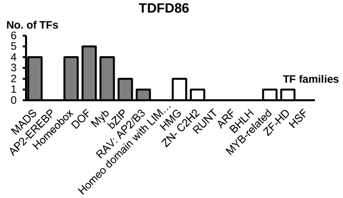

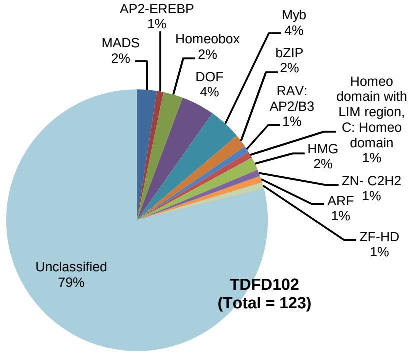

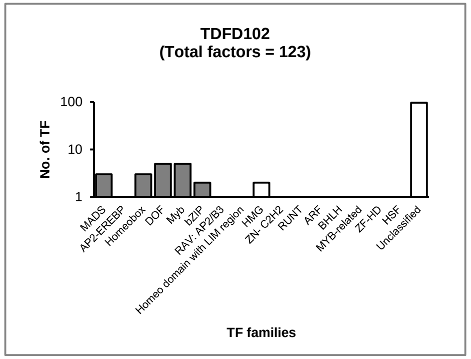

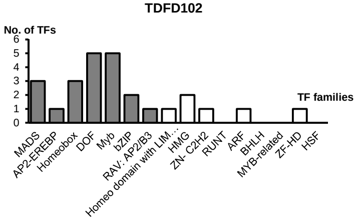

2. List of targets of TDFD142 (Ethylene responsive element binding protein)

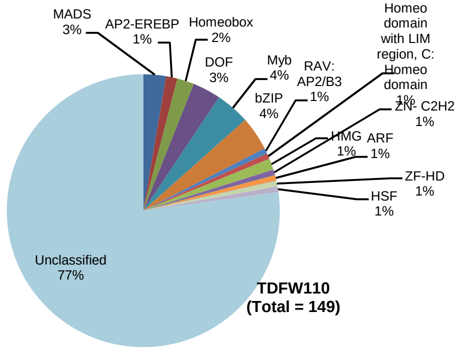

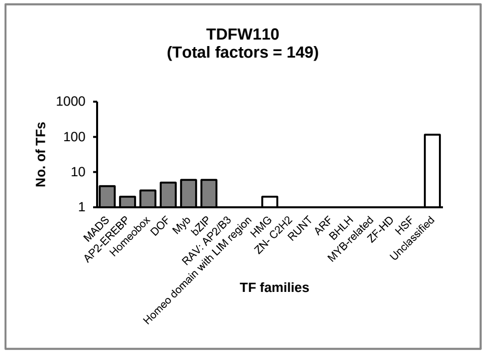

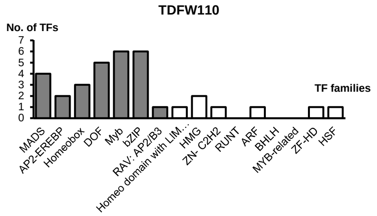

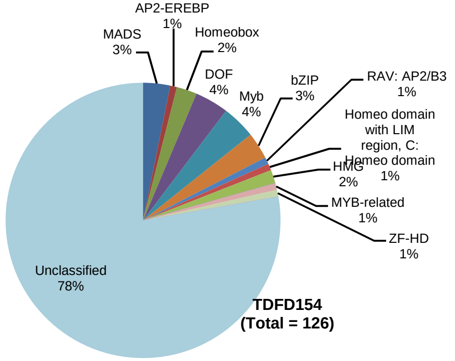

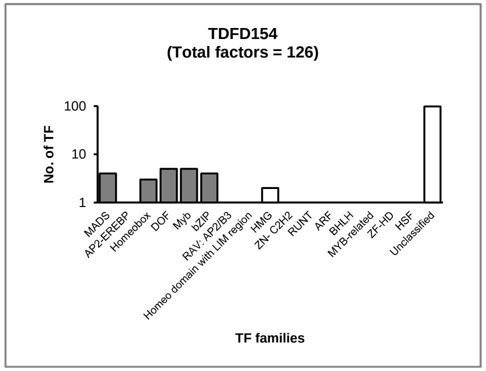

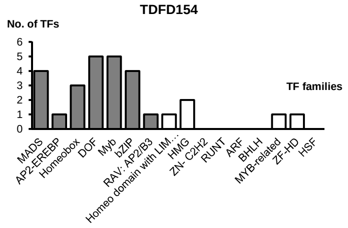

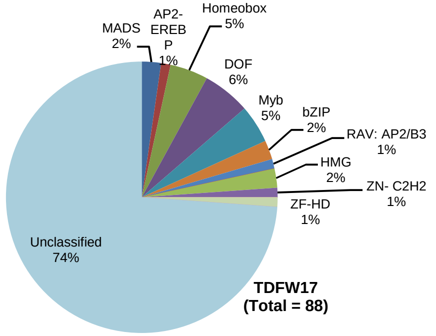

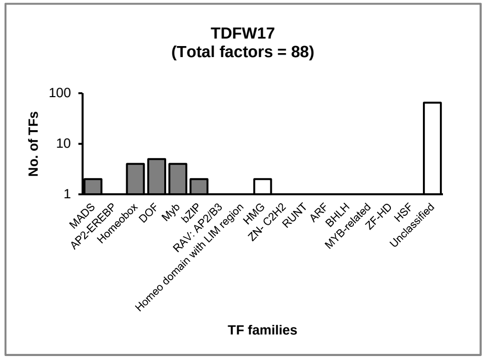

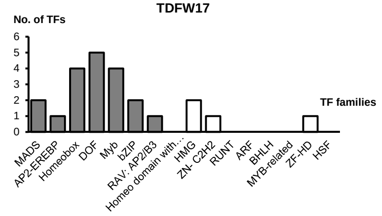

2. List of targets of TDFD142 (Ethylene responsive element binding protein)

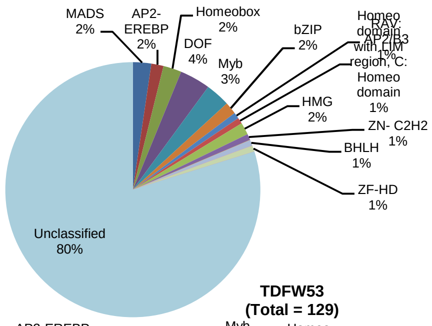

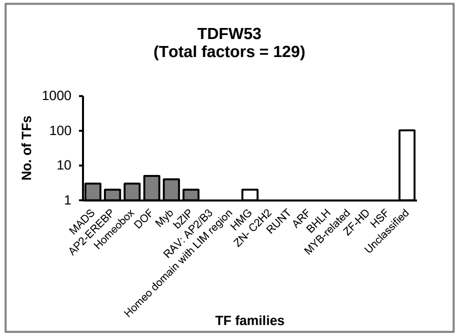

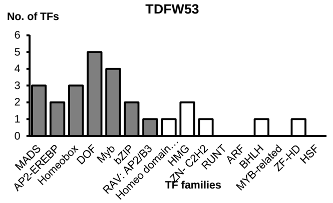

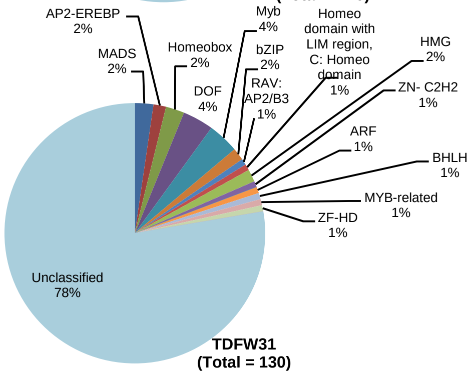

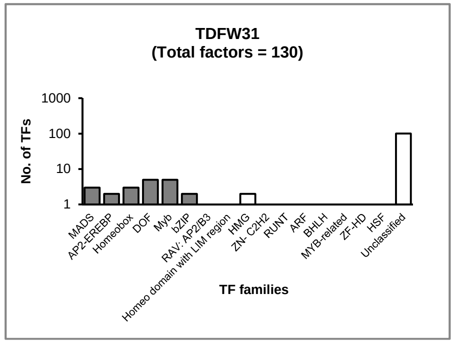

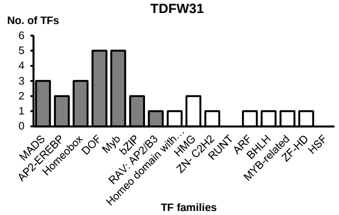

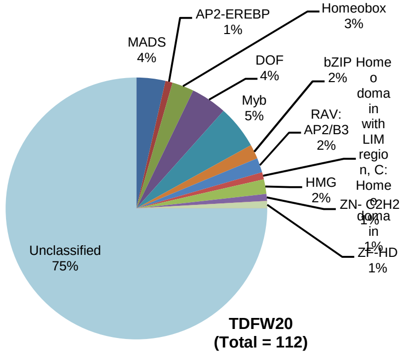

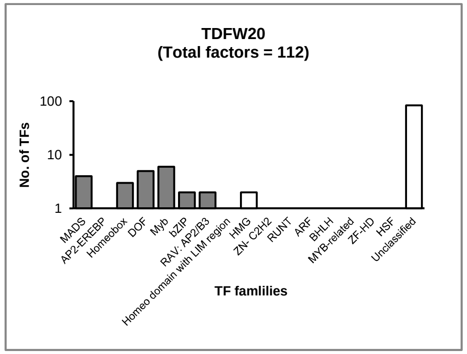

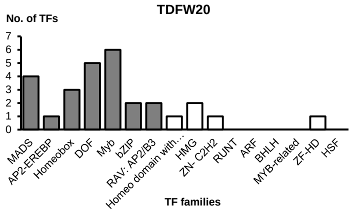

2. List of targets of TDFD142 (Ethylene responsive element binding protein)

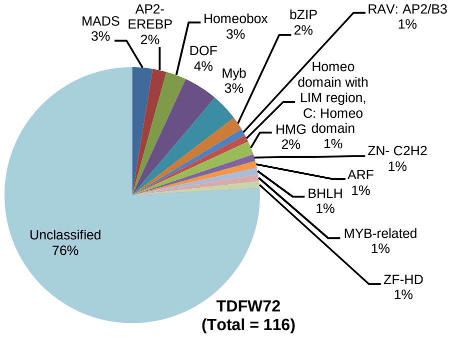

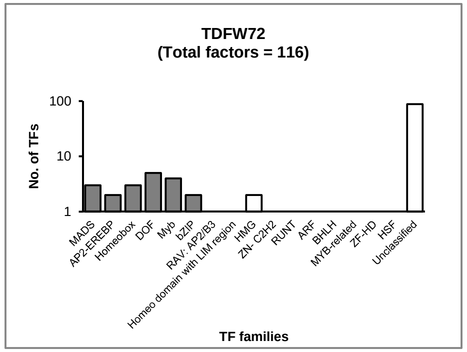

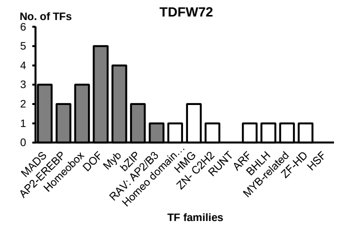

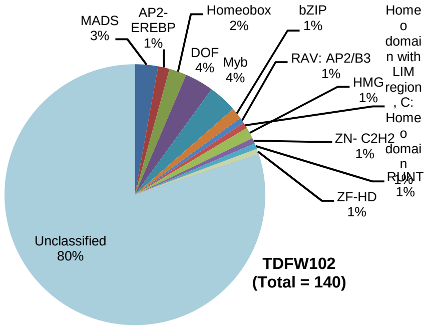

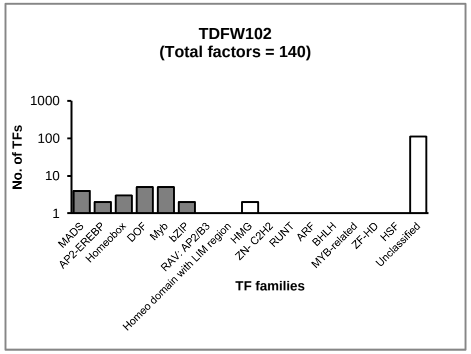

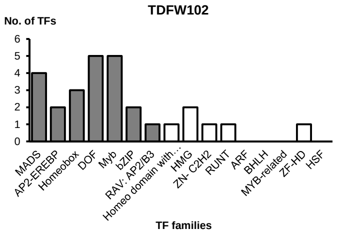

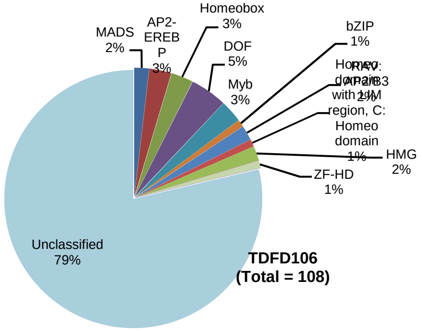

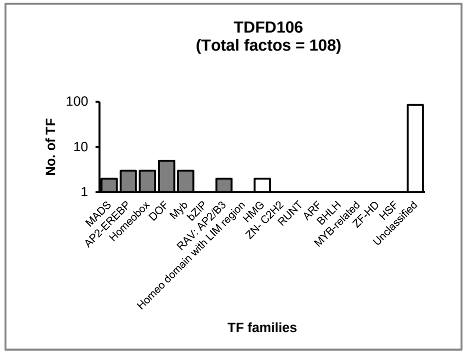

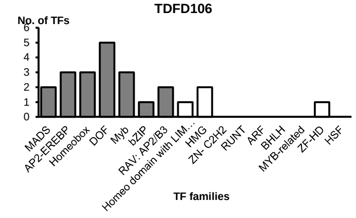

2. List of targets of TDFD142 (Ethylene responsive element binding protein)

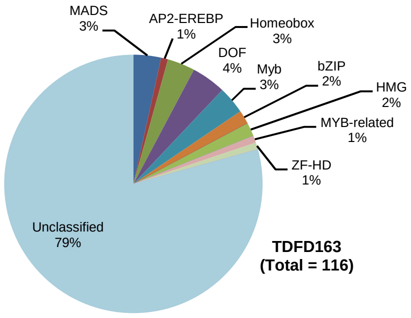

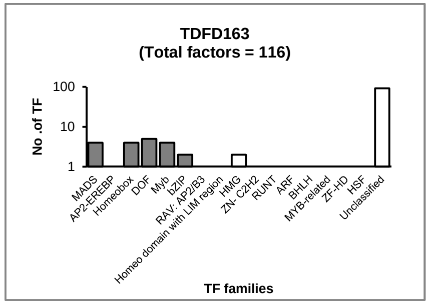

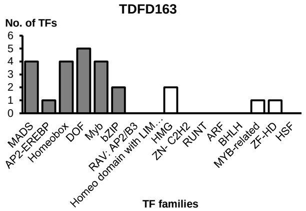

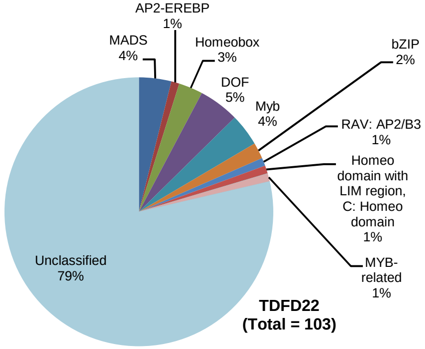

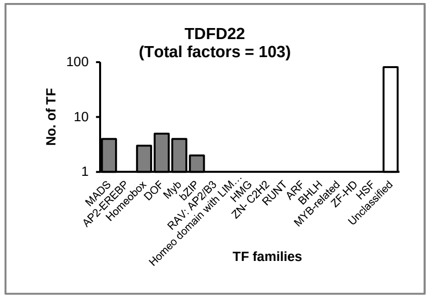

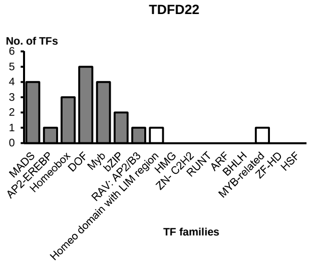

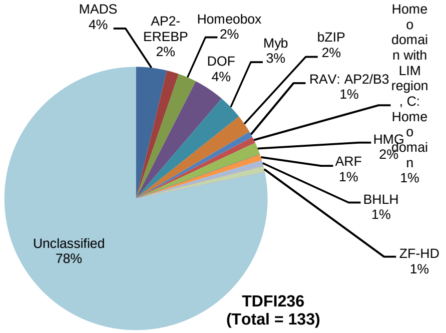

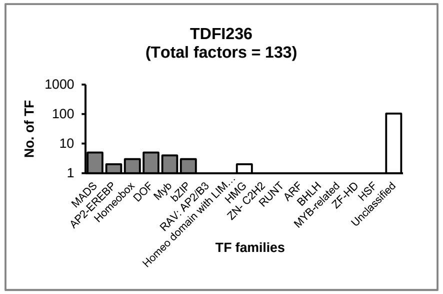

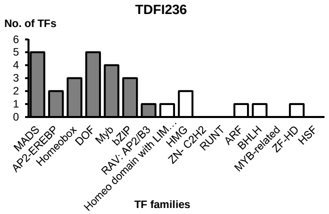

Unique Target found in D142

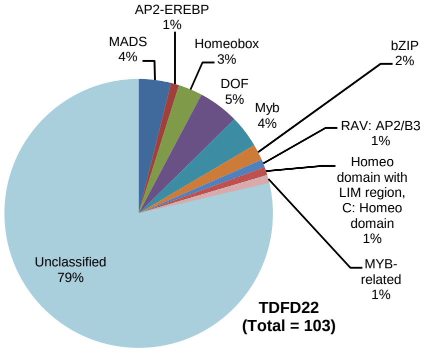

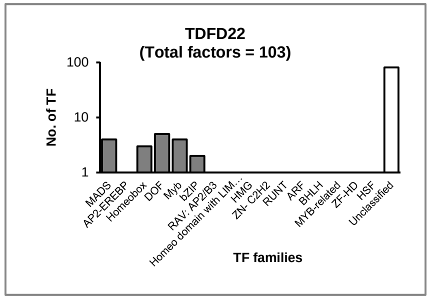

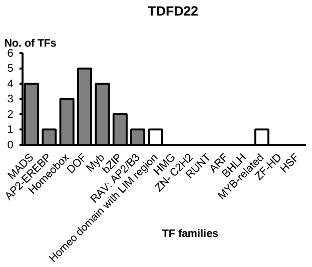

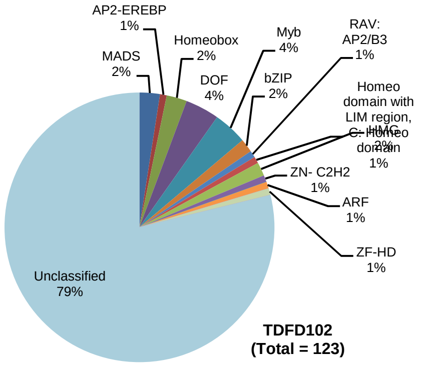

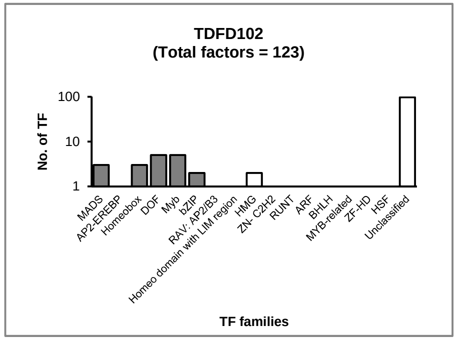

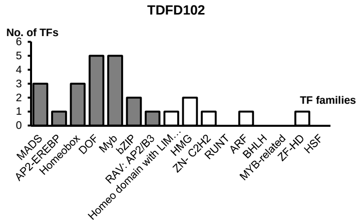

3. List of targets of TDFI236 (WRKY DNA-binding protein 33)

Order	TF	Target
1	I236	D82
2	I236	D106
3	I236	D142
4	I236	W110

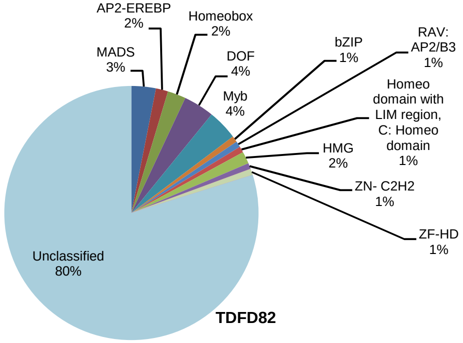

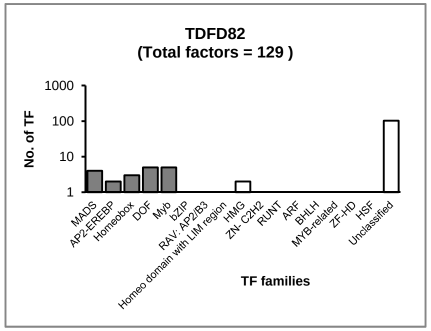

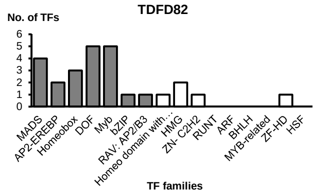

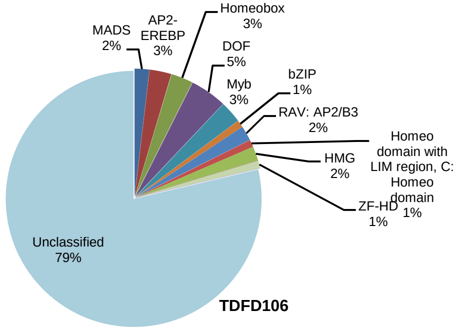

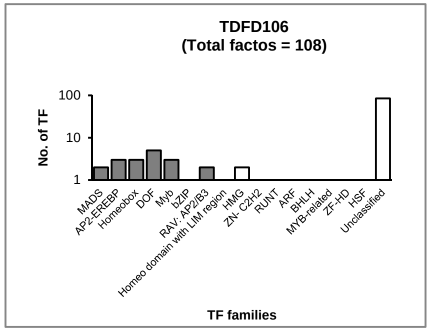

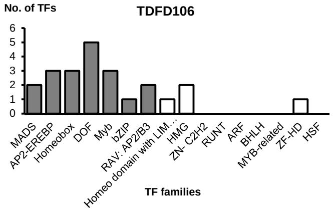

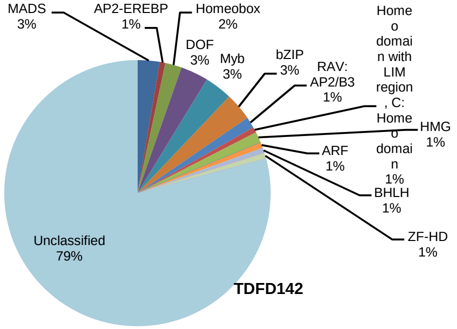

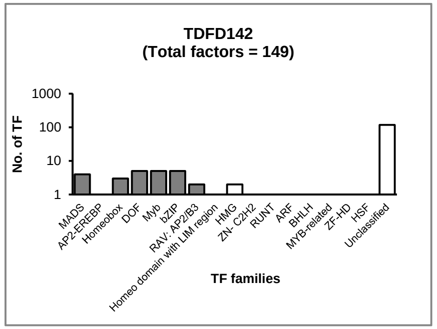

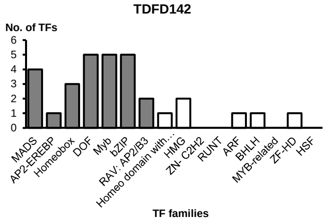

3.List of targets of TDFI236 (WRKY DNA-binding protein 33)

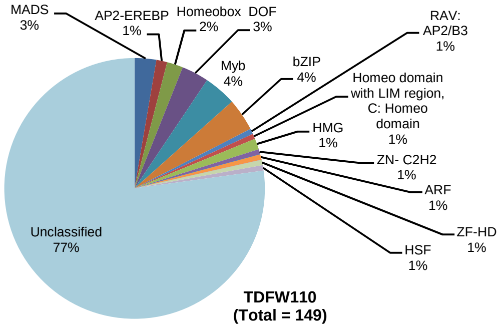

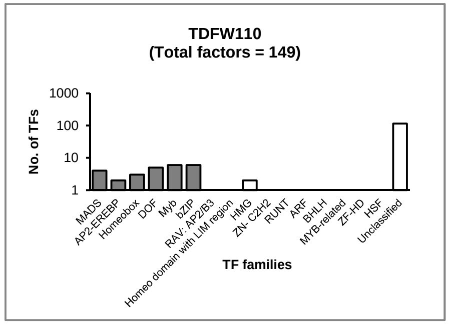

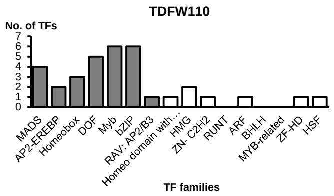

4. List of targets of TDFW17 (Vascular plant one zinc finger protein)

Order	TF	Target
1	W17	D26
2	W17	D82
3	W17	D86
4	W17	D106
5	W17	D142
6	W17	D163
7	W17	W31
8	W17	W102

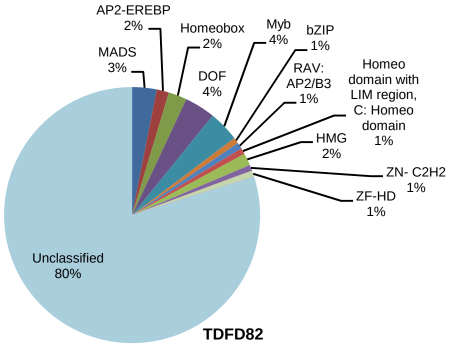

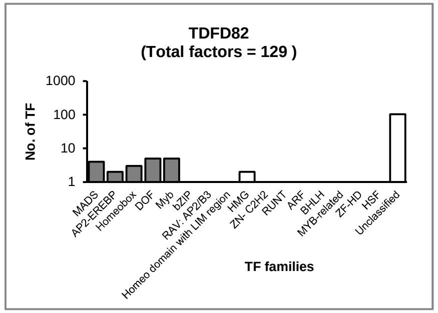

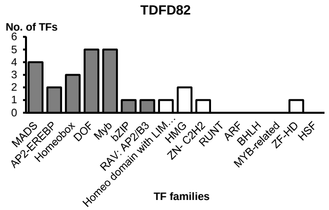

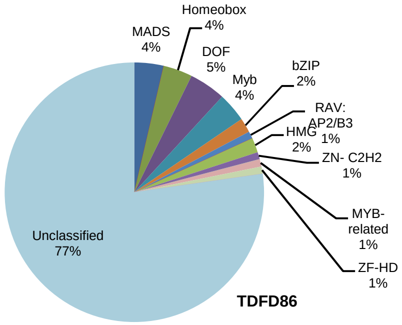

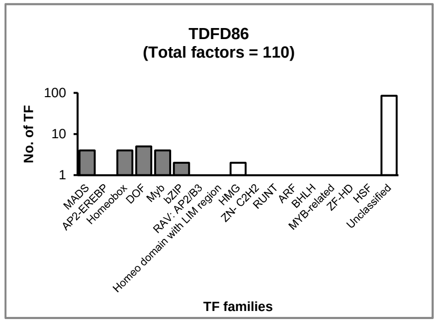

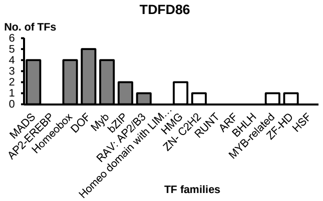

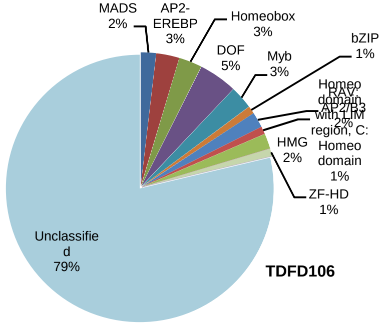

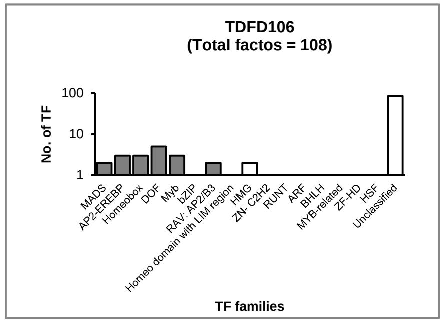

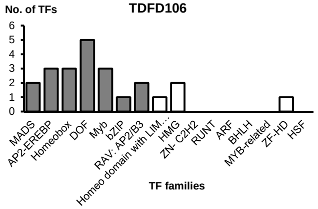

4. List of targets of TDFW17 (Vascular plant one zinc finger protein)

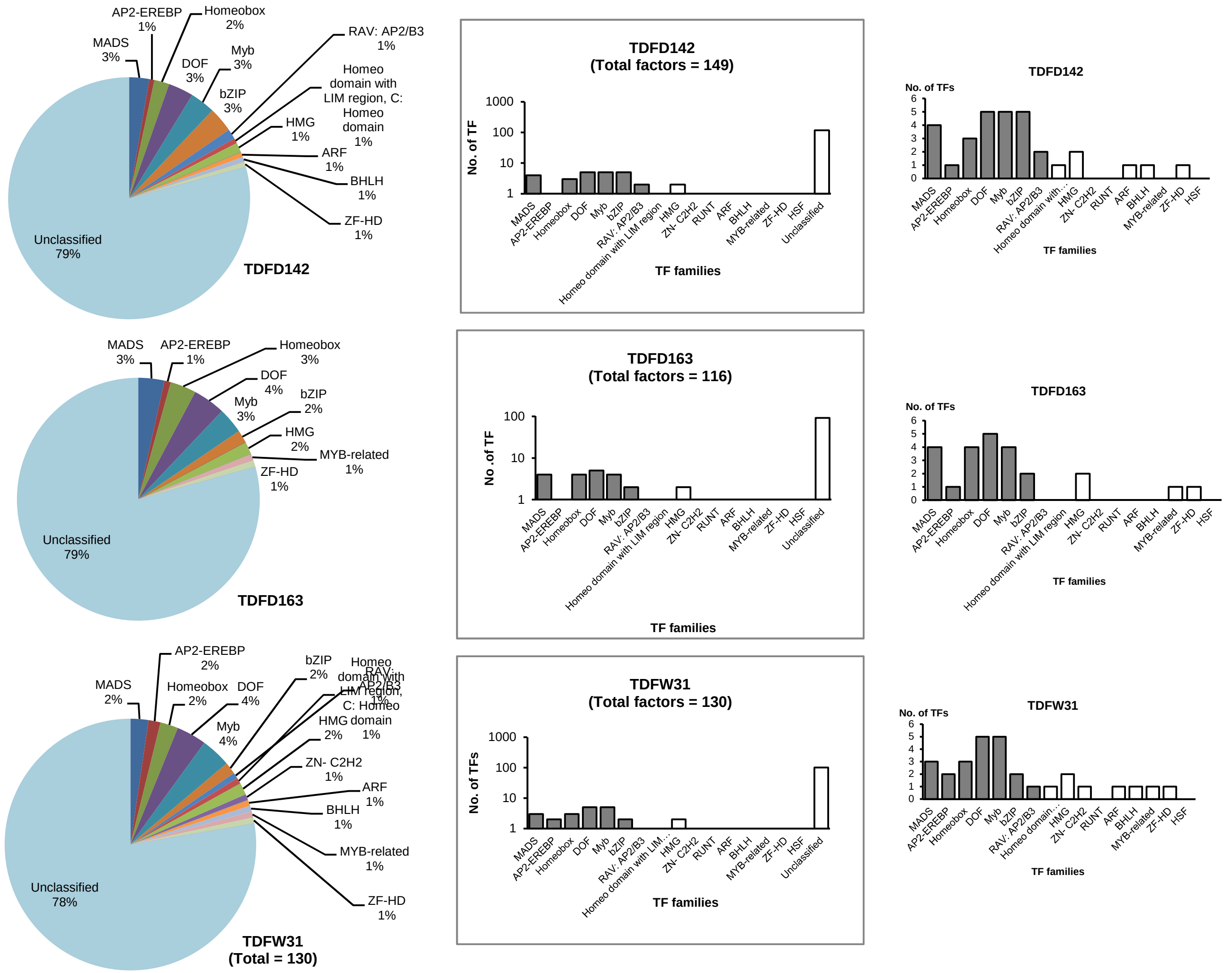

4. List of targets of TDFW17 (Vascular plant one zinc finger protein)

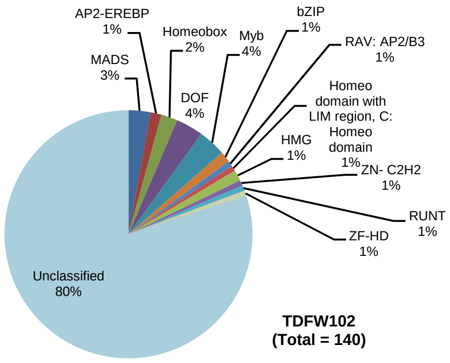

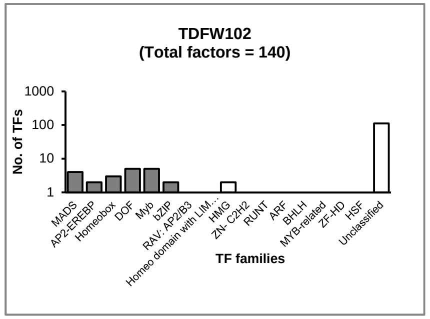

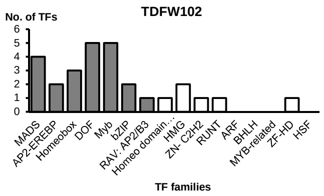

5. List of targets of TDFW20 (Zinc finger family protein)

Order	TF	Target
1	W20	D66
2	W20	D106
3	W20	D142
4	W20	W49
5	W20	W53
6	W20	W72
7	W20	W110
8	W20	W116

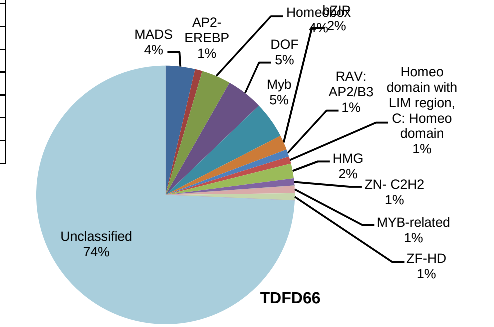

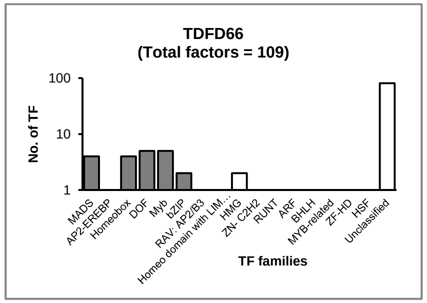

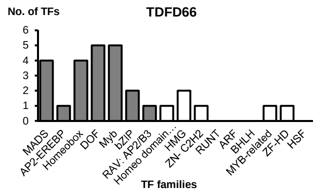

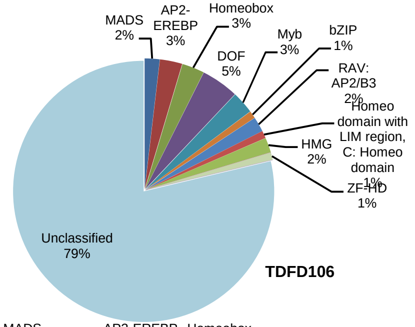

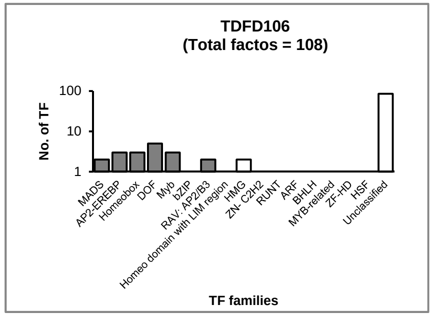

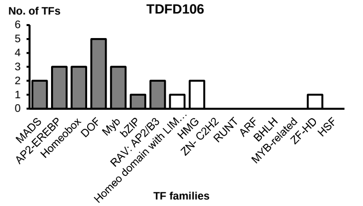

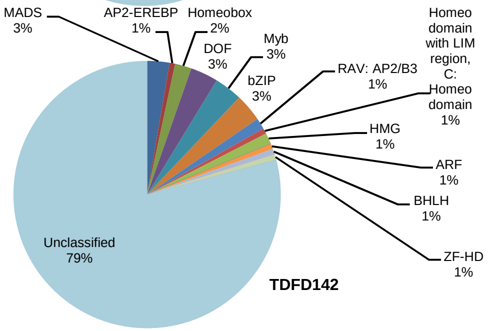

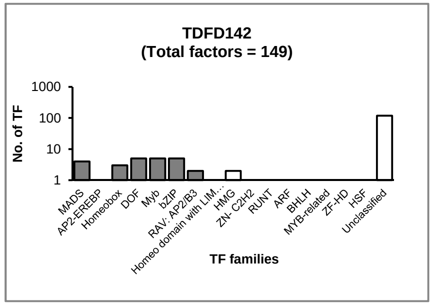

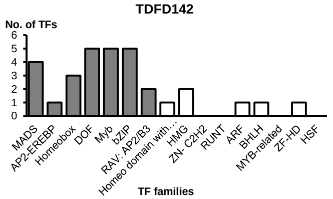

5. List of targets of TDFW20 (Zinc finger family protein)

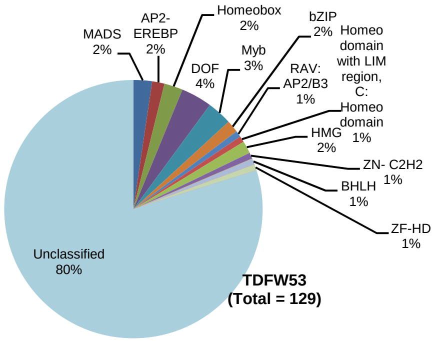

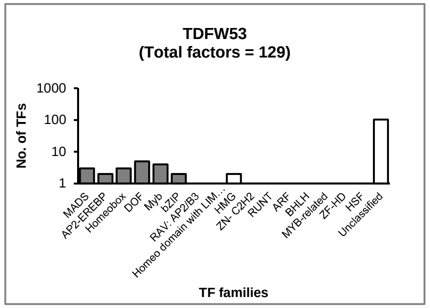

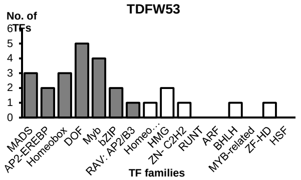

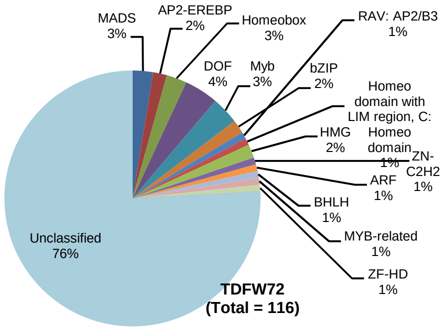

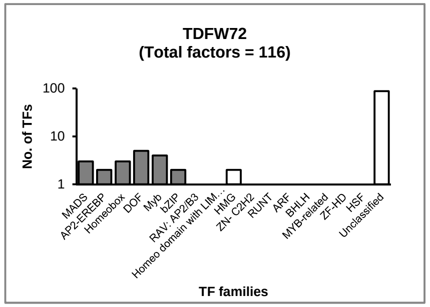

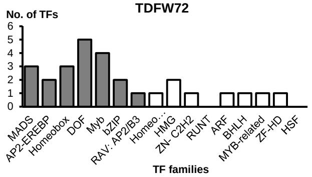

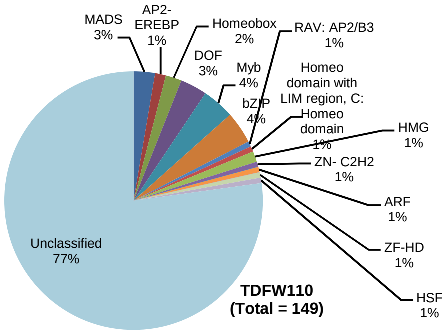

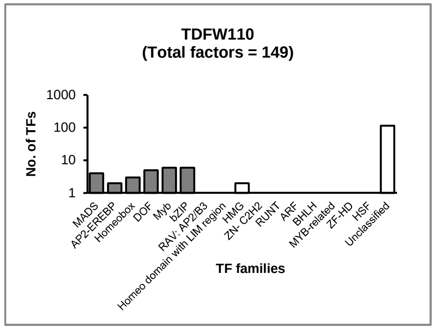

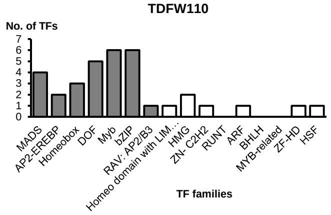

5. List of targets of TDFW20 (Zinc finger family protein)

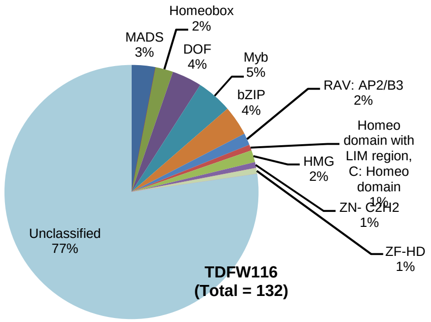

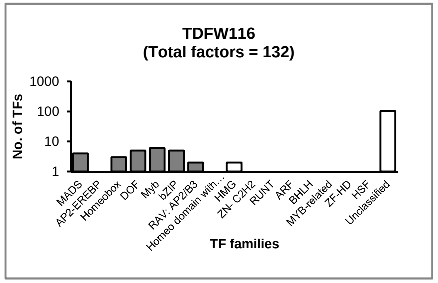

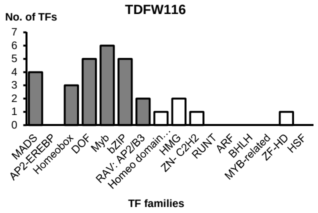

Supplement: S2 File — (PDF) [file pone.0137602.s003.pdf]
